# Attention capture by trains and faces in children with and without autism spectrum disorder

**DOI:** 10.1371/journal.pone.0250763

**Published:** 2021-06-18

**Authors:** Nichole E. Scheerer, Elina Birmingham, Troy Q. Boucher, Grace Iarocci

**Affiliations:** 1 The Brain and Mind Institute, Western University, London, Canada; 2 Department of Psychology, Simon Fraser University, Burnaby, Canada; 3 Faculty of Education, Simon Fraser University, Burnaby, Canada; Bournemouth University, UNITED KINGDOM

## Abstract

This study examined involuntary capture of attention, overt attention, and stimulus valence and arousal ratings, all factors that can contribute to potential attentional biases to face and train objects in children with and without autism spectrum disorder (ASD). In the visual domain, faces are particularly captivating, and are thought to have a ‘special status’ in the attentional system. Research suggests that similar attentional biases may exist for other objects of expertise (e.g. birds for bird experts), providing support for the role of exposure in attention prioritization. Autistic individuals often have circumscribed interests around certain classes of objects, such as trains, that are related to vehicles and mechanical systems. This research aimed to determine whether this propensity in autistic individuals leads to stronger attention capture by trains, and perhaps weaker attention capture by faces, than what would be expected in non-autistic children. In Experiment 1, autistic children (6–14 years old) and age- and IQ-matched non-autistic children performed a visual search task where they manually indicated whether a target butterfly appeared amongst an array of face, train, and neutral distractors while their eye-movements were tracked. Autistic children were no less susceptible to attention capture by faces than non-autistic children. Overall, for both groups, trains captured attention more strongly than face stimuli and, trains had a larger effect on overt attention to the target stimuli, relative to face distractors. In Experiment 2, a new group of children (autistic and non-autistic) rated train stimuli as more interesting and exciting than the face stimuli, with no differences between groups. These results suggest that: (1) other objects (trains) can capture attention in a similar manner as faces, in both autistic and non-autistic children (2) attention capture is driven partly by voluntary attentional processes related to personal interest or affective responses to the stimuli.

## 1. Introduction

From infancy, children must select stimuli to attend to from a vast array of competing information within their environment. Selective attending may occur because certain stimuli are captivating to the infant’s senses (e.g., a flash of light, the sudden sound of a rattle). In this case, low-level features of the visual stimulus such as its colour, size, or orientation lead to automatic, or involuntary, attention capture [1]. As children develop, they voluntarily attend to certain stimuli (e.g., an arrow, a clock), because they provide relevant information to accomplish a particular goal such as finding the playground or knowing when it is time for a treat. The voluntary attention system serves to guide the child’s attention to current task priorities, but also accounts for factors like previous experience with, and the child’s personal interest in, the visual stimulus [2]. Evidence suggests that involuntary mechanisms are the first to develop, with voluntary mechanisms reaching their peak performance by early to mid-adulthood [3,4].

One object that has a strong effect on the prioritization of attention is the human face [5–8]. An attentional bias for faces is present from birth in typically developing infants [9,10], and is thought to drive the specialization of cortical structures for face perception [11]. Given the early emergence of the attentional prioritization of faces, theories emphasizing the evolutionary and biological significance of faces were proposed that suggest that since faces are so essential to our survival, infants have an innate subcortical mechanism that allows for the specialization of cortical areas for face processing [12]. This specialization facilitates the ability of faces to capture attention automatically [13]. Other theories emphasize the role of experience and suggest that the behavioral and neural specialization for faces is a byproduct of considerable experience perceiving and discriminating faces [14–16].

Support for the role of experience in tuning the behavioural and neural specialization of faces comes from studies examining perceptual expertise. These studies found that when experts discriminate amongst exemplars of their category of expertise, in particular categories of homogeneous non-face objects, such as birds, dogs, or novel objects like “Greebles”, face-specific behavioural and neural mechanisms are recruited [14,17,18]. For example, faces are described as being processed holistically, with information across the whole face influencing behaviour to a larger extent than information derived from single features like the eyes or mouth [17,19–21]. Individuals who become experts in a particular class of objects also demonstrate more holistic processing of the object of expertise [22]. Similarly, the fusiform face area (FFA), which typically becomes active while individuals view faces, is also active when observers view non-face objects for which they have a high level of visual expertise [6,23,24]. This evidence suggests that as individuals become more experienced with a class of object, face or otherwise, it changes the way they process these objects both behaviourally and neurally.

In the experimental setting, faces have been found to engage the attention of non-autistic adults more than objects [25–27]. Face stimuli guide eye-movements away from targets [5] and they slow search performance when task-irrelevant [7]. Furthermore, the manual detection of a target is faster when it appears at the location of a face relative to a non-face object location [28, although, see 29,30], suggesting that participants’ attention was spontaneously biased to the face region, thereby facilitating detection of targets subsequently appearing there. Similar results were found in non-autistic children, who demonstrate slower search performance in the presence of task-irrelevant upright [8] and inverted [31] faces. However, this effect of irrelevant faces on task performance was not found for autistic children [8].

A lack of attentional bias toward faces is commonly reported in autistic children [32–35]. Abnormal cortical specialization for face processing has also been reported in autistic individuals [36–39]. Along with the reduced attentional bias to faces, autistic children show an attentional or oculomotor bias towards non-social information [8,40–42], with corresponding neural specialization for non-social, rather than social information [40,42]. It is estimated that over 75% of autistic children possess circumscribed interests [43,44], characterized by an intense and persistent preoccupation with specific objects or object parts [43,45]. The visual attention of autistic children is captured more frequently and sustained longer on objects that are common circumscribed interests relative to faces and other objects [41,46,47]. It is possible that as a result of reduced early social motivation and consequently limited exposure to faces, coupled with increased exposure to objects related to the child’s circumscribed interest, the behaviours and neural circuitry that typically become tuned for faces instead become specialized for non-social objects. Although compelling, this hypothesis is difficult to test due to few available studies comparing attention to faces vs. non-face objects in autistic individuals.

Attention capture tasks are commonly used to determine how various classes of objects influence task performance when included as task-irrelevant distractors. For example, Langton and colleagues (2008) utilized a capture task to examine whether visual target identification was influenced by the presence of a task-irrelevant face distractor. The results indicated that participants were slower to detect a target butterfly among a circular array of objects when a face appeared in the array. Participants performed the target detection task while viewing the stimuli in both an upright and inverted orientation. Inverting the stimuli is thought to prevent configural processing of the stimuli [48,49], which means the participant must instead rely on low-level image based properties such as contrast and edge density that remain unchanged when the stimulus is inverted [7]. Thus, when Langton and colleagues (2008) found attention capture by face distractors only when the stimuli were presented in the upright orientation, this provided support for the idea that the attention capture was driven by a voluntary attentional bias possibly due to the social relevance of the faces, rather than the low-level features of the face stimuli. However, without a key comparison to a non-face category of objects, we do not know whether faces are unique in their ability to capture attention within this paradigm, and whether attention capture for face vs. non-face distractors differs in autistic and non-autistic children.

The aim of Experiment 1 was to examine potential attentional biases to faces and trains in autistic and non-autistic children using the attention capture task previously utilized by Langton and colleagues [7]. Based on the previously reported attentional biases to non-face objects in autistic children and adults, train stimuli served as another class of objects, similar to faces, that have the potential to capture attention, but carry no evolutionary or biological significance. Furthermore, trains are frequently identified as objects of circumscribed interest in autistic children [50,51]. Thus, this study allowed us to examine if the attentional bias for faces previously reported [7], is also present in children, and whether this attentional bias is stronger for trains, relative to faces, in autistic children. Given previous findings [7], we expect that (1) non-autistic, but not autistic children, will be slower to identify the target butterfly when face, relative to train and neutral, distractors are present in an upright orientation, but not when the stimuli are inverted. This would demonstrate greater attention capture by faces in non-autistic, relative to autistic, children. If the faces capture attention more in the upright, relative to the inverted orientation, it would also provide support for the notion that the attention capture is driven by a voluntary attentional bias, rather than low-level features of the face stimuli. We also expect that (2) autistic children will be slower to identify the target butterfly when train, relative to face and neutral, distractors are presented in either orientation, providing support for the notion that the visual attention of autistic children is captured more by objects that are common circumscribed interests, relative to faces and other objects [41,46,47]. Importantly, since the autistic children being recruited for this study are not required to have circumscribed train interests, we do not expect to find the inversion effect often observed when experts perform at task that includes stimuli related to their expertise. While our main measure of attention capture was manual response times (similar to [7,28]), other research groups have used oculomotor measures to demonstrate a spontaneous attention bias for faces within various experimental tasks [5] and that this bias is weaker in autistic individuals (e.g., [33]). We chose to supplement our analysis of manual response times with eye tracking measures to look for additional evidence that faces attract attentional resources, and for our hypothesis that this bias would be weaker in autistic children (and potentially stronger for trains for this group of children). While these analyses are exploratory, we felt that they were important because, while overt measures (eye movements) often converge with manual response measures of attention, they can be dissociated. In addition, some of our recent work suggests that social stimuli may attract early “breakaway” eye movements even though manual response times do not show a spontaneous biasing of attention for faces during a dot-probe task [29]. This previous empirical work motivated our analyses of first fixation latency to the target, fixation count to target, and first fixation latency to the critical distractors. Finally, eye movement research has shown that autistic children show a detail-oriented, perseverative exploration of circumscribed interest objects, relative to non-autistic children [41]. Thus, we also included an analysis of fixation count to critical distractors, to determine if autistic children explored trains more than faces, and more so than non-autistic children. If faces and non-social objects related to circumscribed interests are prioritized by the attentional system of non-autistic and autistic children, respectively, we would expect this prioritization to delay the first fixation to the target, reduce the number of times the target is fixated, decrease the first fixation latency to the prioritized distractor, as well as increase the number of fixations to the prioritized distractor. The aim of Experiment 2 was to assess children’s valence and arousal ratings of the stimuli used in Experiment 1 to determine whether the affective value of these stimuli may be related to their relative attention capture. Given autistic individuals have demonstrated greater exploration and perseveration on particular classes of non-social objects, like trains, over faces [41,46], we expect trains will produce higher valence and arousal ratings for the autistic children, relative to faces and neutral objects. On the other hand, faces readily capture the attention of non-autistic children [8,31], thus we hypothesize that faces will produced higher valence and arousal ratings for non-autistic children, relative to faces and neutral objects.

## 2. General methods

### 2.1 Participants

Eighty-four autistic and non-autistic participants between the ages of 6 and 14 years participated in this experiment. The children were recruited through community advertisements, and the Autism and Developmental Disorder Lab’s database. Participant groups were age matched (see Table 1 for participant information). None of the participants included in experiment 1 or 2 were reported to have any visual or neurological impairments. The parent or guardian provided informed consent for participation. All procedures used in experiment 1 and 2, were approved by the Simon Fraser University Research Ethics Board, and were in accordance with the World Medical Association 2013 Declaration of Helsinki.

**Table 1:** Participant characteristics.

Experiment 1 Manual	Sex	Age (M; SD)	IQ (M; SD)	AQ (M; SD)
Non-Autistic	35 male, 20 female	9.79; 1.69	104.22; 14.92	15.29; 6.86
Autistic	20 male, 9 female	10.35; 1.93	109.07; 12.84	32.15; 5.25
*P*		.17	.13	< .001
*D*		.31	.35	2.76
Experiment 1 Eye-Tracking	Sex	Age (M; SD)	IQ (M; SD)	AQ (M; SD)
Non-Autistic	11 male, 6 female	9.16; 2.24	111.28; 13.50	11.50; 6.28
Autistic	8 male, 2 female	9.02; 2.00	102.11; 13.38	33.78; 6.76
*p*		.864	.090	< .001
*d*		.066	.682	3.415
Experiment 2	Sex	Age (M; SD)	IQ (M; SD)	AQ (M; SD)
Non-Autistic	11 male, 6 female	9.37; 1.66	114.77; 10.35	15.00; 6.81
Autistic	15 male, 3 female	10.06; 1.99	95.18; 17.87	32.06; 5.34
*p*		.272	.001	.001
*d*		.377	1.342	2.788

Note: IQ was measured by the Wechsler Abbreviated Scale of Intelligence (WASI; [52]; AQ = Autism Quotient). The p-values were obtained from independent samples t-tests conducted to compare the age, IQ, and AQ scores across autistic and non-autistic participants. The d-values are effect sizes calculated with Cohen’s d.

### 2.2 Measures

Diagnosis for all autistic participants in both experiment 1 and 2 was confirmed by a British Columbia (BC) clinical diagnostic report along with the Ministry of Child and Family Development ASD funding eligibility report. In the province of BC, substantial government funding is allocated to children with an ASD diagnosis, thus there are standardized diagnostic practices in place. This includes diagnosis by a trained clinician who uses an Autism Diagnostic Observation Schedule (ADOS) and Autism Diagnostic Interview-Revised (ADI-R) and their clinical judgment to determine the diagnosis. To assess current level of autism symptoms, parents completed the Autism Quotient (AQ) parent-report questionnaire [53]. As expected, AQ scores for participants in the autistic group were significantly higher than those for the participants in the non-autistic group (see Table 1).

## 3. Experiment 1 methods

### 3.1 Apparatus and stimuli

In order to optimize data collection while managing the limitations imposed by only being able to eye-track a single participant at a time, a group of participants took part in a version of the experiment in which only manual responses were measured (Non-Autistic: n = 38, Autistic: n = 21), while the remainder of the participants completed the task while their eye movements were also tracked (Non-Autistic: n = 25, Autistic: n = 10).

The participants in the manual-only version of the task were seated 60 cm away from a Dell OptiPlex 7010 computer with a 23” monitor (1920 x 1080 pixel resolution), running E-Prime 2.0 software (Psychology Software Tools, Pittsburgh, PA) for stimulus presentation.

Eye-tracking participants were seated 60 cm away from a Thermaltake PC with a 17” Acer V173 monitor (1280 x 1024 pixel resolution), running Experiment Builder software (SR Research, Ltd, Hamilton, Ontario, Canada) for stimulus presentation. Left eye movements were tracked using a desktop mounted Eyelink 1000 eye tracker (SR Research), with an accuracy of 0.5° of visual angle and sampling rate of 1000 Hz. Participants were seated in a dark room with their head supported on a chin rest to ensure a fixed distance from the display computer screen. A calibration procedure was conducted prior to the experiment. Drift correction was also performed at the beginning of each trial, by having participants fixate a dot in the center of the computer screen and press the spacebar. Each trial required fixation calibration within 0.3° of the pre-trial fixation in order to proceed. Only fixation events were analyzed. In our study, the online saccade detector of the eye tracker was set to detect saccades with an amplitude of at least 0.1°, using an acceleration threshold of 8000°/s^2^ and a velocity threshold of 30°/s. Eye blinks were not analyzed in our study. Regions of interest (ROIs) were 3 cm by 3 cm regions around the stimuli, subtending approximately 2.86 by 2.86 degrees of visual angle. All stimuli, experimental blocks, and experimental procedures were identical to the manual-only version of the task, except for the addition of eye-tracking.

**Fig 1:**
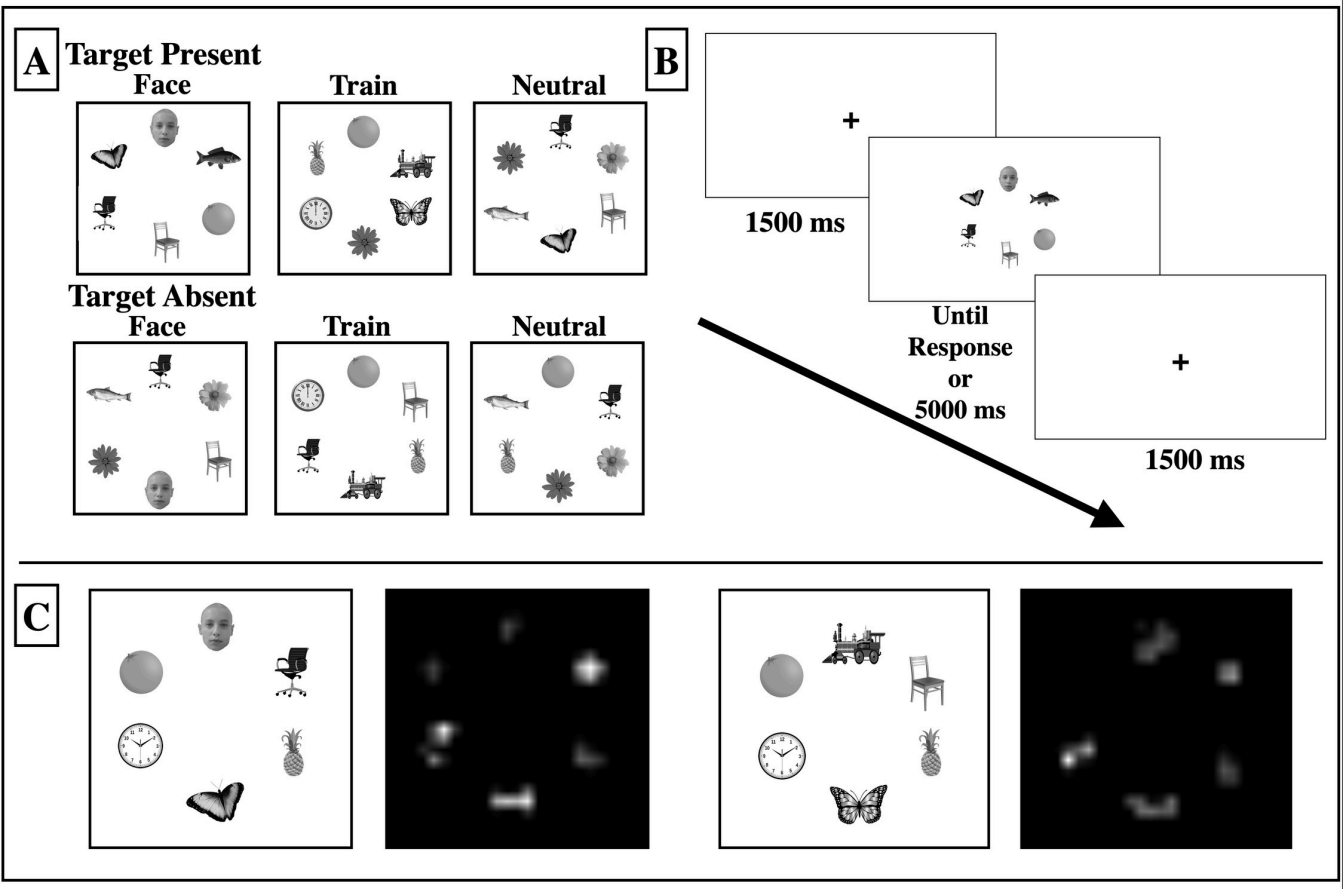
Experimental setup. A: Participants viewed an array of 8 grey-scale objects on a white background. Target present arrays contained a butterfly stimulus, while face and train arrays contained critical face and train distractors. The remaining objects occupying the arrays were classified as neutral stimuli (i.e. fruit, flower, chair, clock, and fish stimuli). B: On each trial a fixation cross appeared in the centre of the screen for 1500 ms. A stimulus array was then presented and participants were instructed to indicate as quickly and accurately as possible whether a butterfly target was present. Search displays remained on screen until participants made a keypress or until 5000 ms had elapsed. C: Sample visual saliency maps generated using Saliency Toolbox [55,56] in Matlab; to remain consistent with other users of this toolbox we used all default parameters (Iterative Normalization). Note: due to copyright restrictions, many of the images in this figure are representative. See S1 File for information on how to access the actual images used in the experiment.

The stimuli consisted of grey-scale objects on a white background (see Fig 1). Objects were different shapes, but were sized to fit within a 3 cm x 3 cm square. Objects were presented in a circular array, with each object 4.3° of a visual angle from the central point of the display. Six different pictures of butterflies were used as target objects, while distractor objects were eight fruit, flower, chair, clock, fish, face, and train exemplars. The distractors of special interest in this study were faces, of which there were eight different exemplars (four male and four female), which had neutral expressions and were cropped to remove the external features (hair and ears). Face stimuli were borrowed with permission from Langton and colleagues (2008). A second category of special interest distractors, trains, were included in addition to the previously mentioned categories of targets and distractors. Train stimuli were created by taking photographs of model trains and removing the background using photoshop. Face and train stimuli were of equal mean luminance contrast (face, M = 40.57; train, M = 43.47, *t*(14) = .97, *p* = .35), as computed with the SHINE toolbox ([54]). Representative stimuli and resultant arrays, including saliency maps computed with the Saliency Toolbox [55,56] are shown in Fig 1C).

On each experimental trial, stimulus arrays were randomly generated by the experimental software. *Target-Present* arrays contained a butterfly exemplar randomly located in one of the object positions along with 5 distractor exemplars, while *Target-Absent* arrays contained 6 distractor exemplars. Distractors were randomly chosen from the neutral stimulus categories without replacement. In order to determine the influence of face and train distractors on target identification, one third of all trials (both target-present and target-absent) contained a face distractor amongst randomly selected neutral distractors, one third of all trials contained a train distractor amongst randomly selected neutral distractors, and the last one third of all trials only contained randomly selected neutral distractors (see Fig 1).

In order to assess whether attention capture was influenced by low-level stimulus features, half of the stimulus arrays were presented in an upright orientation, while the other half were presented in an inverted orientation. Orientation was blocked and counterbalanced across participants such that half of the participants were presented with upright stimulus arrays first, while the other half of the participants were presented with inverted stimulus arrays first.

### 3.2 Procedure

On each trial a fixation cross appeared in the centre of the screen for 1500 ms. A stimulus array was then presented and participants were instructed to indicate as quickly and accurately as possible whether a butterfly target was present. The “1” and “0” keys on a standard keyboard were used to indicate “target present” and “target absent”, respectively. A butterfly sticker was placed over the “1” key, while a “X” sticker was placed over the “0” key to remind the child of the appropriate keys to press. Search displays remained on screen until participants made a keypress, or until 5000 ms had elapsed. No mention of faces or trains was made during the instructions. Participants started with 12 practice trials, which were not included in the statistical analyses. Participants then completed two blocks (one upright, one inverted block) of 120 experimental trials, with a break provided between blocks. The entire procedure took approximately 20 minutes.

### 3.3 Data analysis

For each participant, the mean percentage of correct trials (accuracy), as well as mean and median response times (RTs) were calculated for all 12 experimental conditions (target: present, absent, x distractor: train, face, neutral, x orientation: upright, inverted). While mean RTs were useful for identifying outliers, median RT was ultimately used for statistical analyses as it is often a more representative measure of the centre of a distribution. Any trials where RT was more than 3 standard deviations from the condition mean were considered outliers and removed. In addition, any participant whose mean accuracy and/or RT was more than 3 standard deviations from the group mean were removed. Seven non-autistic and one autistic participant were excluded as their responses were considered too slow based on this criteria. In addition, due to software malfunction, one autistic participant only took part in the upright version of the experiment.

In order to quantify participants’ gaze patterns, *fixation count* and *first fixation time* were calculated and averaged over trials for each condition. To assess eye-movements to the various experimental stimuli, regions of interest (ROI) were created around each stimulus position. *Fixation count* was then calculated as the total number of fixations that landed in each ROI. For trials on which no fixation occurred in the ROI, the variable was assigned a value of zero and was included in the mean calculation. *First fixation time* was calculated as the latency of the first fixation to enter the ROI. For trials on which no fixation occurred in the ROI, the variable was assigned a value of NaN and not included in the mean calculation. Fixation count and first fixation time were calculated for the target ROI (on target present trials) and for distractor ROIs (on both target present and target absent trials).

## 4. Experiment 1 results

Four-way mixed measures analysis of variances (MM-ANOVAs) examined the influence of orientation (upright, inverted), target condition (absent, present), distractor condition (train, face, neutral), and group (autistic, non-autistic) on both accuracy and median RTs. Follow-up Bonferroni pairwise and independent samples t-tests were conducted where applicable. Here, we index an attention capture effect as a slowing of RTs in the presence of a critical distractor (e.g., train and face conditions) relative to when that distractor was absent (neutral condition). To take a closer look at the effect of the distractor conditions, a three-way MM-ANOVA was conducted to examine the influence of target condition (absent, present), distractor condition (train, face), and group (autistic, non-autistic) on both accuracy and median RTs, separately for the upright and inverted orientations. These results can be found in S2 File.

### 4.1 Manual accuracy

**Fig 2:**
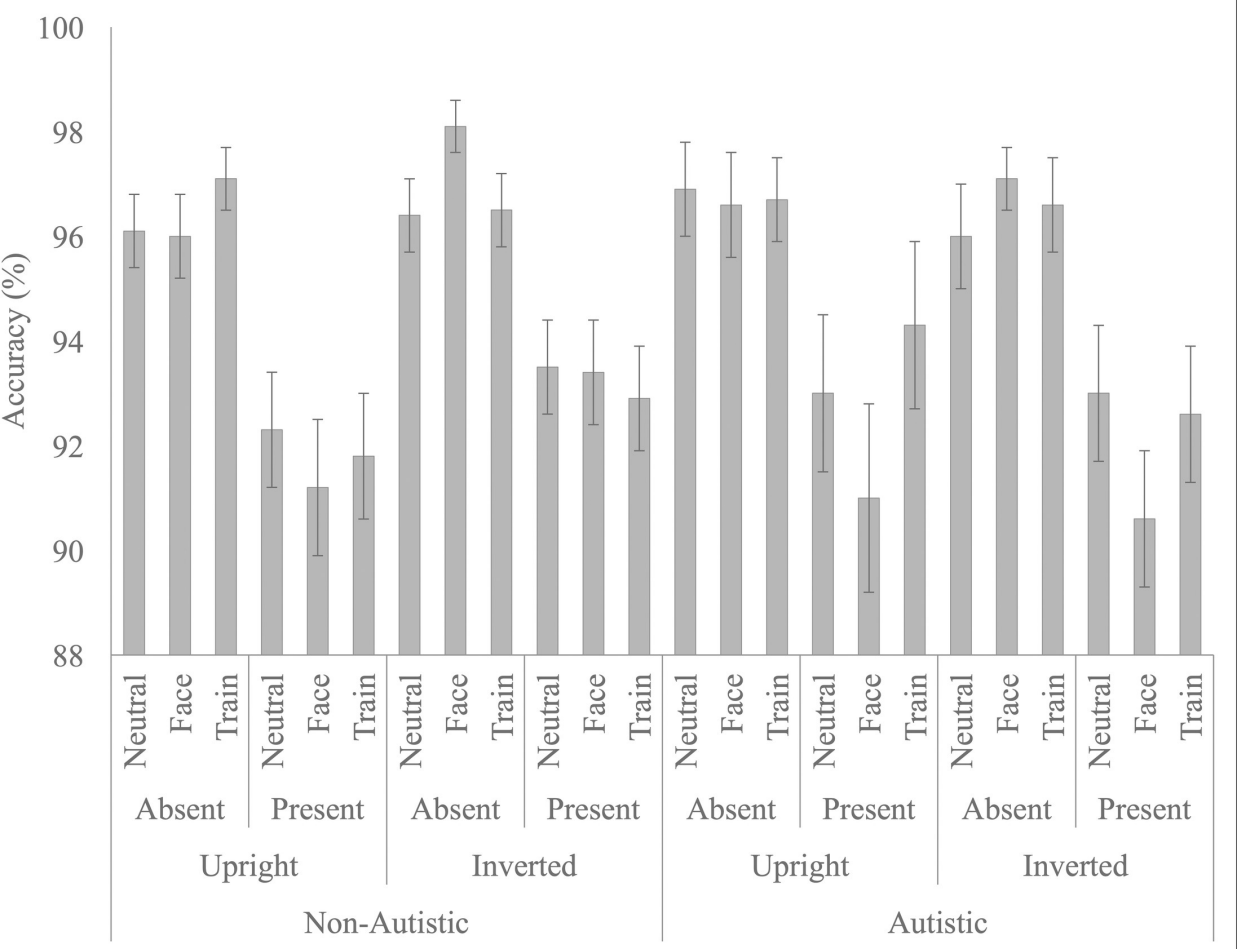
Accuracy (mean and standard error) as a function of diagnostic group (autistic, non-autistic), orientation (upright, inverted), target status (present, absent), and distractor (neutral, train, face).

There was a significant main effect of target, F (1, 73) = 78.204, p < .001, n_p_^2^ = .517, as accuracy was higher when the target was absent, relative to when the target was present (see Fig 2). The interaction between target and distractor was also significant, F (2, 146) = 3.266, p = .041, n_p_^2^ = .043. The main effects of orientation, distractor, and group, as well as all other interactions were not significant.

Follow-up RM-ANOVAs were conducted to investigate the effect of distractor condition on accuracy separately for target absent and target present trials. However, despite the above interaction, there was no effect of distractor condition on accuracy for target present (p = .165) or absent trials (p = .367).

### 4.2 Manual median RT

**Fig 3:**
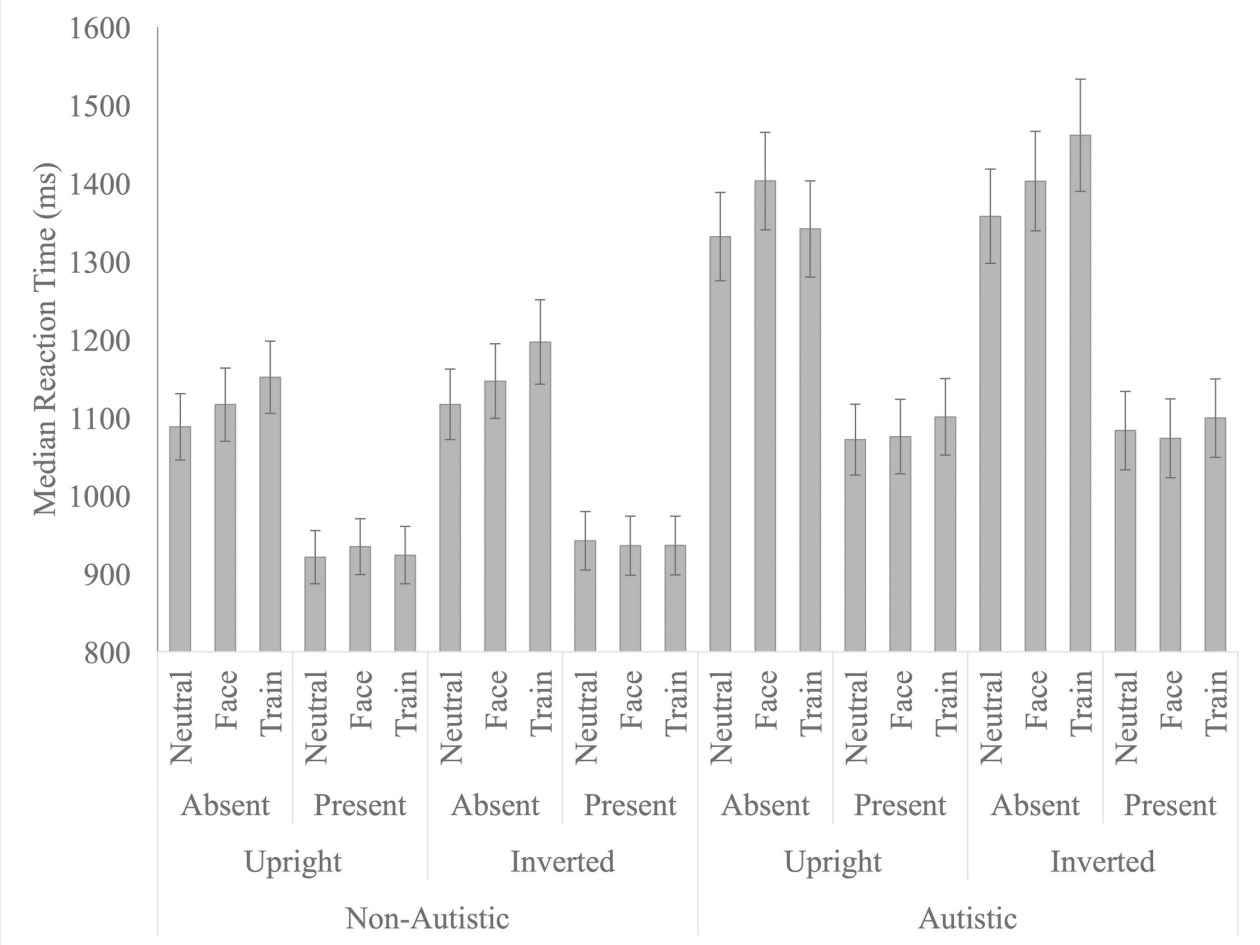
Median reaction time (mean and standard error) for both autistic and non-autistic participants across the upright and inverted stimulus conditions as a function of target (present, absent) and distractor (neutral, train, face).

There was a significant main effect of target, F (1, 73) = 263.351, p < .001, n_p_^2^ = .783, as RT was faster on target present (M = 1007.996, SE = 27.660), relative to absent (M = 1259.298, SE = 35.430) trials. The main effect of group was significant, F (1, 73) = 10.446, p = .002, n _p_^2^ = .125, as autistic children were slower (M = 1233.278, SE = 49.321) than non-autistic children (M = 1034.016, SE = 36.991). The target by group interaction was also significant, F (1, 73) = 9.451, p = .003, n _p_^2^ = .115. Although both groups were faster to respond on target present than absent trials (autistic: t(26) = 9.877, p < .001, d = 1.13; non-autistic: t(47) = 12.848, p < .001, d = 0.77; see Fig 3), autistic children showed a larger mean difference in RT than non-autistic children (autistic: M = 298.909, SE = 30.26; non-Autistic: M = 203.695, SE = 15.854).

**Fig 4:**
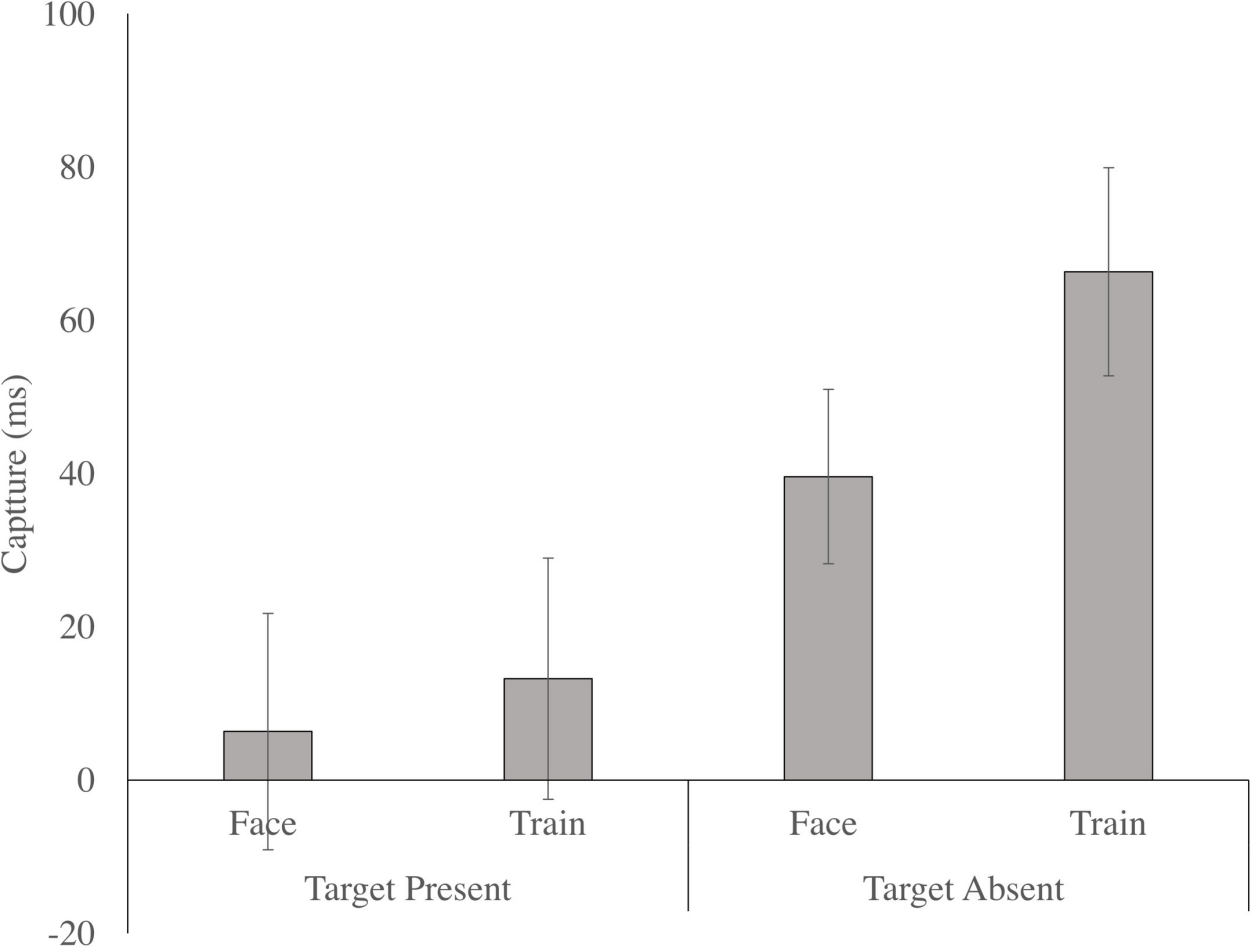
Attention Capture (mean and standard error) by Trains (train-neutral) and Faces (face-neutral) conditions but plotted separately for target present and target absent trials.

The main effect of distractor was significant, F (2, 146) = 9.061, p < .001, n _p_^2^ = .110, as RTs were slower in the face (M = 1135.846, SE = 32.429), and train (M = 1151.213, SE = 32.650) conditions, relative to the neutral condition (M = 1113.881, SE = 29.573; p = .033 and p < .001, respectively). To directly compare attention capture by trains versus faces, we computed a difference score (attention capture effect) by subtracting participants’ median RT in the neutral condition from their median RTs in the train (M = 36.70, SE = 8.13) and face (M = 20.43, SE = 8.06) distractor conditions. A paired samples t-test indicated that trains had a marginally larger attention capture effect relative to faces, t(74) = 1.802, p = .076, d = 0.23, 95% CI [-0.09, 0.56 (see Fig 4). The distractor by group interaction, F(2, 146) = .195, p = .823, n _p_^2^ = .003, and the orientation by distractor by group interaction, F(2, 146) = 1.019, p = .364, n _p_^2^ = .014, were both not significant. Given our a priori hypothesis that attention capture by trains and faces would differ across autistic and non-autistic children, independent samples t-tests were conducted comparing capture by trains (neutral–train distractor conditions) and capture by faces (neutral–face distractor conditions) across the autistic and non-autistic children. Capture by trains, t(73) = .266, p = .808, d = 0.06, [-0.41, 0.54], and capture by faces, t(73) = .651, p = .563, d = 0.15, [-.032, 0.64], did not differ across the autistic and non-autistic children.

The target by distractor interaction was significant, F (2, 146) = 5.628, p = .004, n _p_^2^ = .072, as when the target was present, RTs were not significantly different across the distractor conditions, F (2, 148) = .331, p = .719, n _p_^2^ = .004. However, when the target was absent, F (2, 148) = 11.660, p < .001, RTs were slower in the face (M = 1229.022, SE = 38.378), and train (M = 1255.750, SE = 39.096), relative to the neutral condition (M = 1189.437, SE = 34.387; p = .003 and p < .001, respectively). To directly compare attention capture by trains versus faces on target absent trials, we again computed a difference score (attention capture effect) for the train and face conditions relative to the neutral condition. While the attention capture effect was numerically larger for the train condition (M = 66.31, SE = 13.59) than for the face condition (M = 39.59, SE = 11.40), a paired samples t-test indicated that this difference was not statistically significant, t(74) = 1.663, p = .101, d = 0.25, [-0.08, 0.57].

The orientation by target interaction was significant, F (1, 73) = 5.061, p = .027, n_p_^2^ = .065, as the mean RT difference between upright and inverted stimuli was not significant for target present trials (M = 8.351, SE = 19.976); t(74) = .418, p = .677, d = 0.03, [-0.29, 0.36], but approached significance for target absent trials (M = 39.671, SE = 203.530; t(74) = 1.688, p = .096, d = 0.12, [-0.20, 0.44]; see Fig 3). The main effect of orientation and all other interactions were not significant.

## 5. Discussion manual results

The accuracy and median RT results from Experiment 1 indicate that across upright and inverted stimulus presentation conditions, autistic and non-autistic children were more accurate, but slower, at determining the presence or absence of the butterfly target when the target was absent. When considering the effect of the different classes of distractor stimuli, these different classes of stimuli did not influence the accuracy of target identification. However, when comparing median RTs across the different distractor conditions, although autistic children were slower overall, they were no less susceptible to attention capture by faces than their age- and IQ-matched non-autistic peers. In addition, we failed to find a distractor by orientation interaction, thus we did not find evidence of an inversion effect for either autistic or non-autistic children. Given previous research has shown that faces are prioritized by the attentional systems of non-autistic individuals when presented in an upright, but not an inverted manner, these findings were unexpected. These manual effects demonstrate that trains and faces had a similar effect on attention capture. Thus, these results do not support the notion that faces are prioritized in the attentional system of children [8,31]. Our data does not support the hypotheses that (1) faces are highly likely to capture the attention of non-autistic children relative to other classes of stimuli, and (2) autistic children are less susceptible to attention capture by faces, or more susceptible to attention capture by trains. Although the manual response data suggests that faces are not prioritized by the attentional system of children, we utilized eye-tracking technology on a subset of the participants (see Table 1 for participant characteristics for this sample) to measure whether overt responses are biased towards faces, or other objects available to the visual system.

## 6 Eye-tracking results

### 6.1 Fixations to target

The butterfly target was fixated at least once on the majority of upright trials (M = 89%, SE = 3.0%) and inverted trials (M = 78%, SE = 1.7%). Independent samples t-tests confirmed that the number of trials on which the target was fixated, did not differ across autistic and non-autistic groups for upright, t (31) = .116, p = .908, d = 0.05, [-0.78, 0.88], nor inverted, t (33) = .261, p = .795, d = 0.13, [-.66, .86], conditions.

### 6.2 First fixation latency to target

Two-way RM-ANOVAs examined the influence of distractor condition, and group on the latency to first fixate the target ROI. Follow-up Bonferroni pairwise and independent samples t-tests were conducted where applicable.

#### 6.2.1 Upright

The main effect of distractor, group, and the distractor by group interaction, were not significant (all p’s > .05).

#### 6.2.2 Inverted

**Fig 5:**
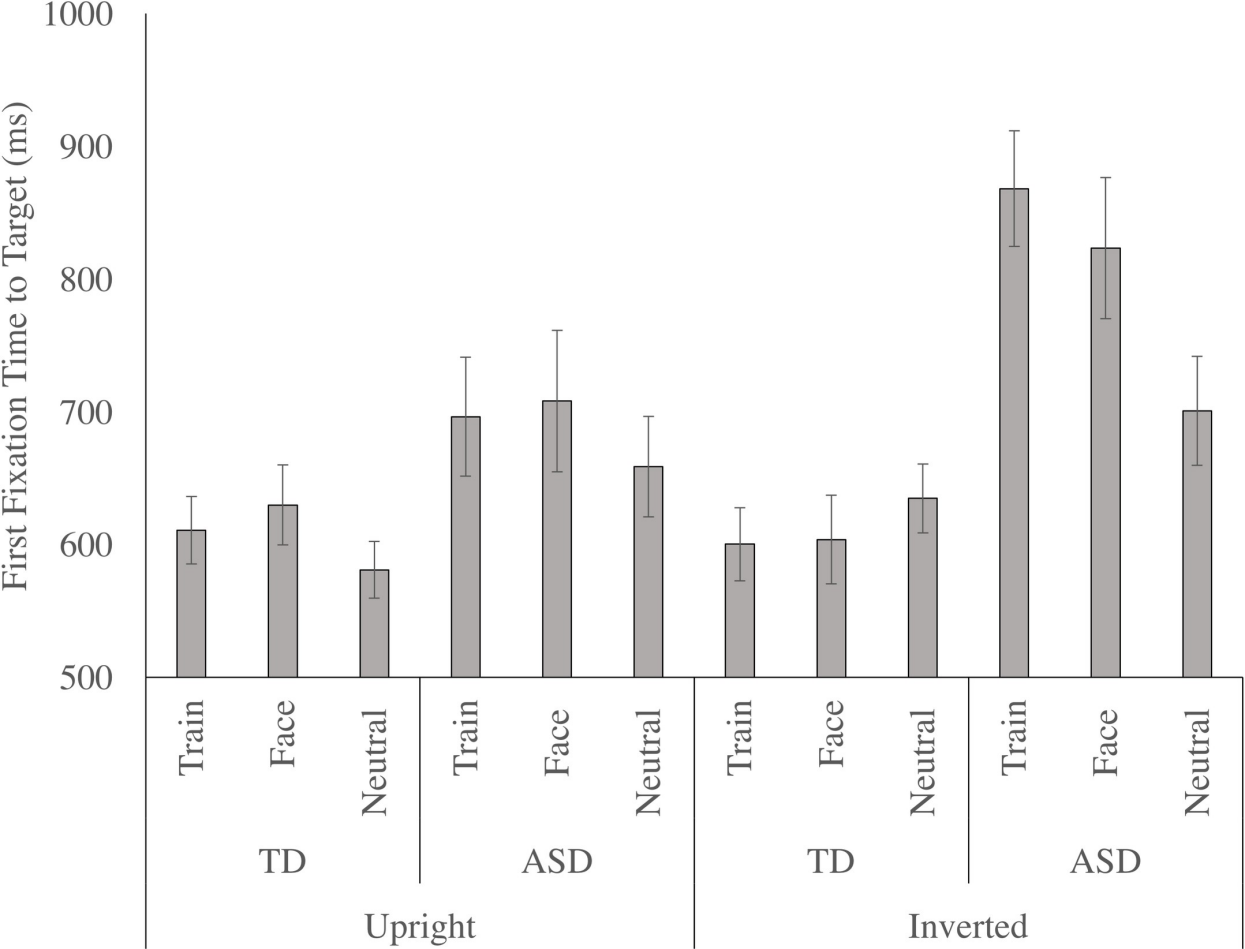
The time elapsed between the beginning of the trial and the first fixation to the target, or the first fixation time to target (mean and standard error), for autistic and non-autistic children in the upright and inverted stimulus conditions as a function of distractor (neutral, train, face).

The main effect of distractor condition, F (2, 66) = 3.916, p = .025, n _p_^2^ = .106, was significant, as the first fixation to the target occurred much slower in the train condition, relative to the neutral condition (p = .003). The face distractors showed a marginal effect on first fixation latency to the target relative to the neutral condition (p = .076). Face and train conditions did not differ (p = 1.000). The main effect of group, F (1, 33) = 15.464, p < .001, n _p_^2^ = .319, was significant as the non-autistic children first fixated the target faster than the autistic children. In addition, a significant distractor by group interaction, F (2, 66) = 9.418, p < .001, n _p_^2^ = .222, indicated that the effect of the distractor condition was largely driven by the autistic children (see Fig 5). Separate RM-ANOVAs indicated that for the non-autistic children, the main effect of distractor was not significant. In contrast, for the autistic children, the main effect of distractor, F (2, 18) = 4.370, p = .028, n _p_^2^ = .327, was significant, with pairwise comparisons showing that first fixation time was slowed by the train distractor, relative to the neutral condition (p = .009); yet only marginally slowed by the face distractor, relative to the neutral condition (p = .054). However, latency of the first fixation to the target was not significantly different between face and train conditions in the autistic children (p = .532).

### 6.3 Fixation count to target

Two-way RM-ANOVAs were conducted to investigate the influence of distractor condition (neutral, train, face), and group (autistic, non-autistic) on the number of fixations to the target ROI, separately for upright and inverted stimulus conditions. Follow-up Bonferroni pairwise and independent samples t-tests were conducted were applicable.

#### 6.3.1 Upright

The main effect of distractor, F (2, 62) = .292, p = .748, n _p_^2^ = .009, group, F (1, 31) = .236, p = .630, n _p_^2^ = .008, and the distractor by group interaction, F (2, 62) = .191, p = .827, n _p_^2^ = .006, were non-significant.

#### 6.3.2 Inverted

The main effect of distractor, F (2, 66) = 3.903, p = .025, n _p_^2^ = .106, was significant as the target was fixated less frequently in the presence of train distractors (M = 1.091, SE = .068), relative to face distractors (M = 1.238, SE = .078; p = .028), but not neutral distractors (M = 1.220, SE = .078; p = .111). The number of fixations to the target ROI did not differ between face and neutral distractor conditions (p = 1.000). The main effect of group, F (1, 33) = .874, p = .357, n _p_^2^ = .026, and the distractor by group interaction, F (2, 66) = .501, p = .608, n _p_^2^ = .015, were not significant.

### 6.4 Fixations to critical distractors

We chose not to analyze fixations in the neutral condition because a) there were no critical distractors present in that condition and b) neutral distractors outnumbered the critical distractors, which would inflate dependent variables such as fixation count. Overall, critical distractors (face, train) were fixated on a minority (<50%) of trials. Paired samples t-tests were utilized to determine whether the percentage of trials where the critical distractor was fixated differed across conditions. For the upright condition, the results indicated that there was a significant effect of distractor condition, t(33) = 7.717, p < .001, d = 1.24, [0.75, 1.81], as there were more trials where the train was fixated (M = 40%, SE = 2.3%), relative to the face (M = 25%, SE = 1.9%). Similarly, for the inverted condition, the results indicated that there was a significant effect of distractor condition, t(35) = 7.102, p < .001, d = 0.91, [0.44, 1.43], as there were more trials where the train was fixated (M = 42%, SE = 3.0%), relative to the face (M = 26%, SE = 2.6%).

### 6.5 First fixation latency to critical distractor

Three-way RM-ANOVAs examined the influence of target condition (absent, present), distractor condition (train, face), and group (autistic, non-autistic) on the latency of the first fixation to the critical distractor ROI. Follow-up Bonferroni pairwise and independent samples t-tests were conducted were applicable.

#### 6.5.1 Upright

**Fig 6:**
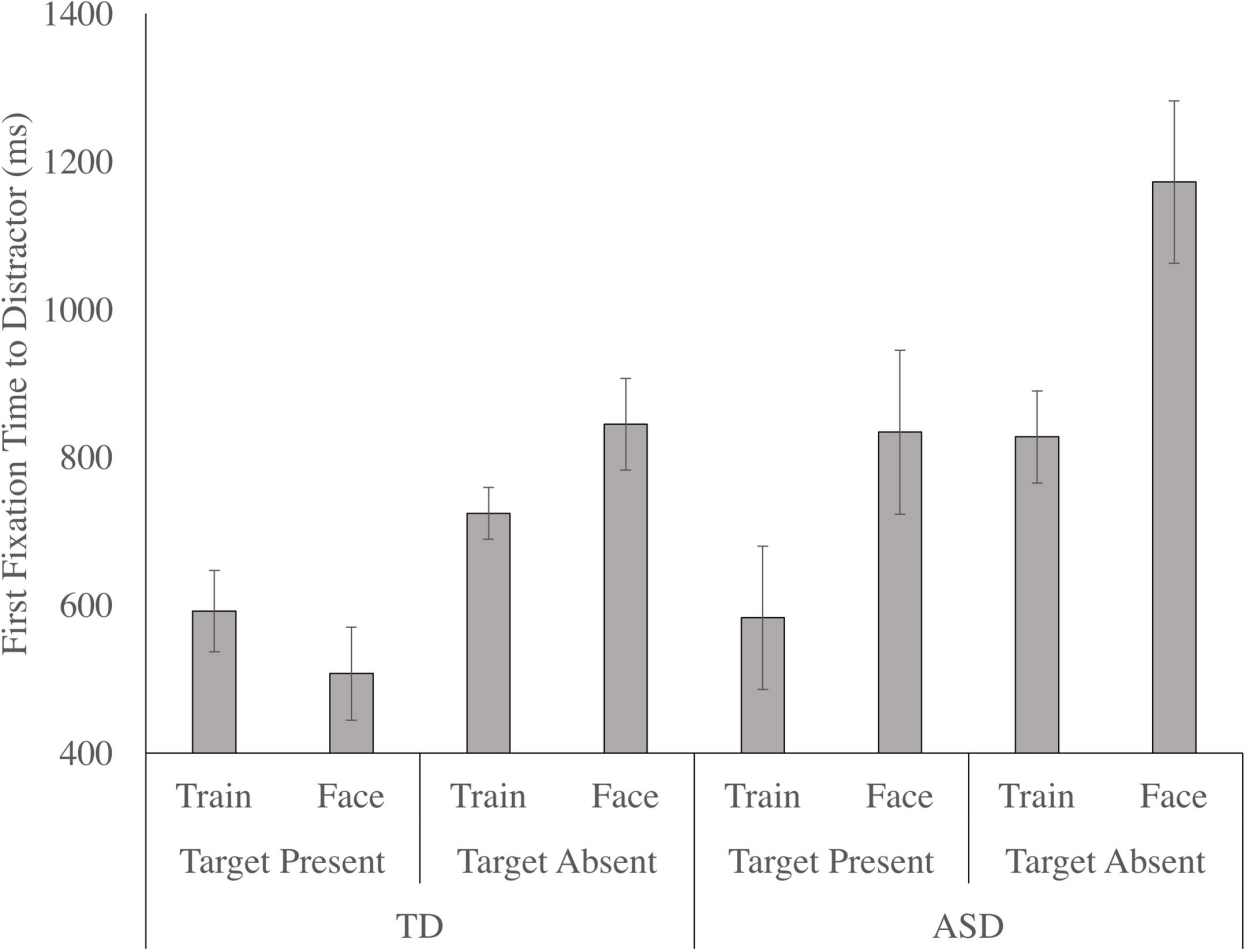
The time elapsed between the beginning of the trial and the first fixation to the distractor, or the first fixation time to distractor (mean and standard error), for both the autistic and non-autistic children in the upright condition as a function of target (present, absent) and distractor (train, face).

There was a significant main effect of target, F (1, 31) = 26.414, p < .001, n _p_^2^ = .460, as the critical distractor was first fixated faster when the target was present, relative to when the target was absent. The main effect of distractor condition was significant, F (1, 31) = 7.301, p = .011, n _p_^2^ = .191, as participants were faster to first fixate the critical distractor when it was a train, relative to when it was a face. However, this effect of distractor condition was largely driven by the autistic group, as reflected in a significant distractor by group interaction, F (1, 31) = .017, p = .898, n _p_^2^ = .001. Follow up paired samples t-tests confirmed that autistic children first fixated the train faster than the face (t (7) = 3.155, p = .016, d = 1.11, [0.07, 2.38]), whereas the non-autistic children showed no difference in the latency to fixate the train and the face. There was a main effect of group, F (1, 31) = 7.993, p = .008, n _p_^2^ = .205, as the non-autistic children first fixated the critical distractors faster than the autistic children (see Fig 6). However, the target by group, as well as the target by distractor, and the target by distractor by group interactions were not significant.

#### 6.5.2 Inverted

There was a significant main effect of target, F (1, 27) = 17.878, p < .001, n _p_^2^ = .398, as the critical distractor was first fixated faster when the target was present, relative to when the target was absent. The main effects of distractor condition, group, and the interactions between target and group, distractor and group, and target and distractor, were not significant. However, the target by distractor by group interaction was significant, F (1, 27) = 10.456, p = .003, n _p_^2^ = .279. Follow up RM-ANOVAs examined the influence of target condition and distractor for each group separately. For the non-autistic children, there was a main effect of target, F (1, 19) = 17.718, p < .001, n _p_^2^ = .483, as first fixation time to critical distractors was faster when the target was present. Although the main effect of distractor condition was not significant, the target by distractor interaction was, F (1, 19) = 4.890, p = .039, n _p_^2^ = .205, with paired samples t-tests confirming that first fixation time to critical distractors did not differ across distractor conditions in the target present condition. However, trains were first fixated faster than faces in the target absent condition, t (22) = 2.202, p = .038, d = 0.68, [0.10, 1.32]. For the autistic children, there was a marginal main effect of target, F (1, 8) = 5.062, p = .055, n _p_^2^ = .388, as first fixation time to critical distractors was faster when the target was present. Although the main effect of distractor was not significant, the target by distractor interaction was, F (1, 8) = 5.405, p = .049, n _p_^2^ = .403, with paired t-tests confirming that (opposite to the pattern observed in the non-autistic children) trains were first fixated faster in the target present condition, t (9) = 3.315, p = .009, d = 0.64, [-0.24, 1.69], but there was no effect of distractor condition in the target absent condition.

### 6.6 Fixation count to critical distractors

Three-way RM-ANOVAs examined the influence of target condition (absent, present), distractor condition (train, face), and group (autistic, non-autistic) on the number of fixations to the critical distractor ROI. Follow-up Bonferroni pairwise and independent samples t-tests were conducted were applicable.

#### 6.6.1 Upright

**Fig 7:**
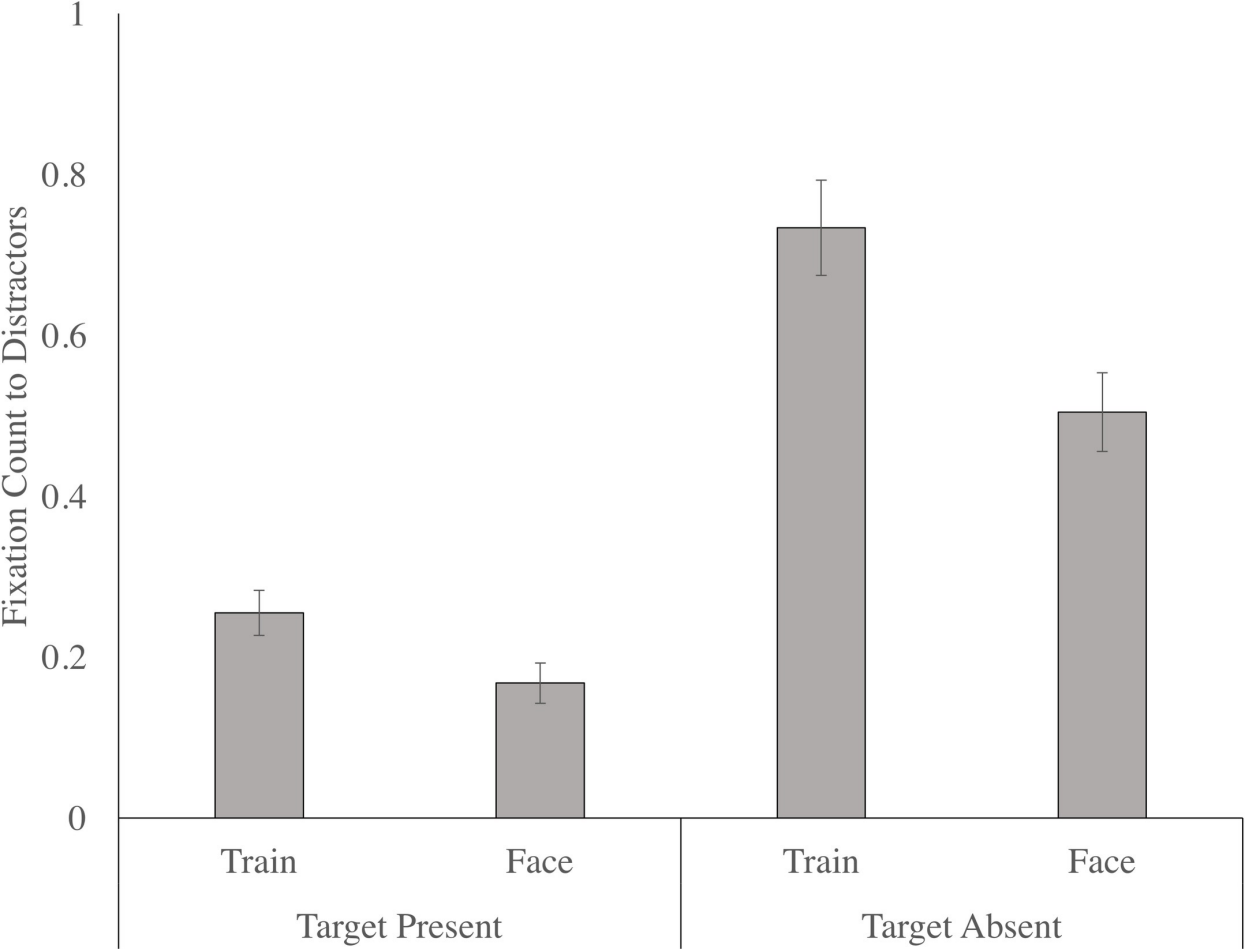
The number of fixations to the distractors (mean and standard error), collapsed across both autistic and non-autistic children in the upright condition as a function of target (present, absent) and distractor (train, face).

There was a significant main effect of target condition, F (1, 31) = 88.195, p < .001, n _p_^2^ = .740, as there were more fixations to the critical distractor ROIs when the target was absent, relative to when the target was present. The main effect of distractor condition was also significant, F (1, 31) = 23.949, p < .001, n _p_^2^ = .436, as participants allocated more fixations to the critical distractor in the train condition, relative to the face condition. This was qualified by a significant target by distractor interaction, F (1, 31) = 6.784, p = .014, n2 = .180, with paired t-tests confirming that train distractors were fixated more than face distractors on target present, t (32) = 2.775, p = .009, d = 0.57, [0.08, 1.09], and target absent, t (32) = 5.528, p < .001, d = 0.89, [.38, 1.41] trials; however, the difference was more pronounced when the target was absent (see Fig 7). The main effect of group, as well as the target by group, distractor by group, and target by distractor by group, interactions were not significant.

#### 6.6.2 Inverted

**Fig 8:**
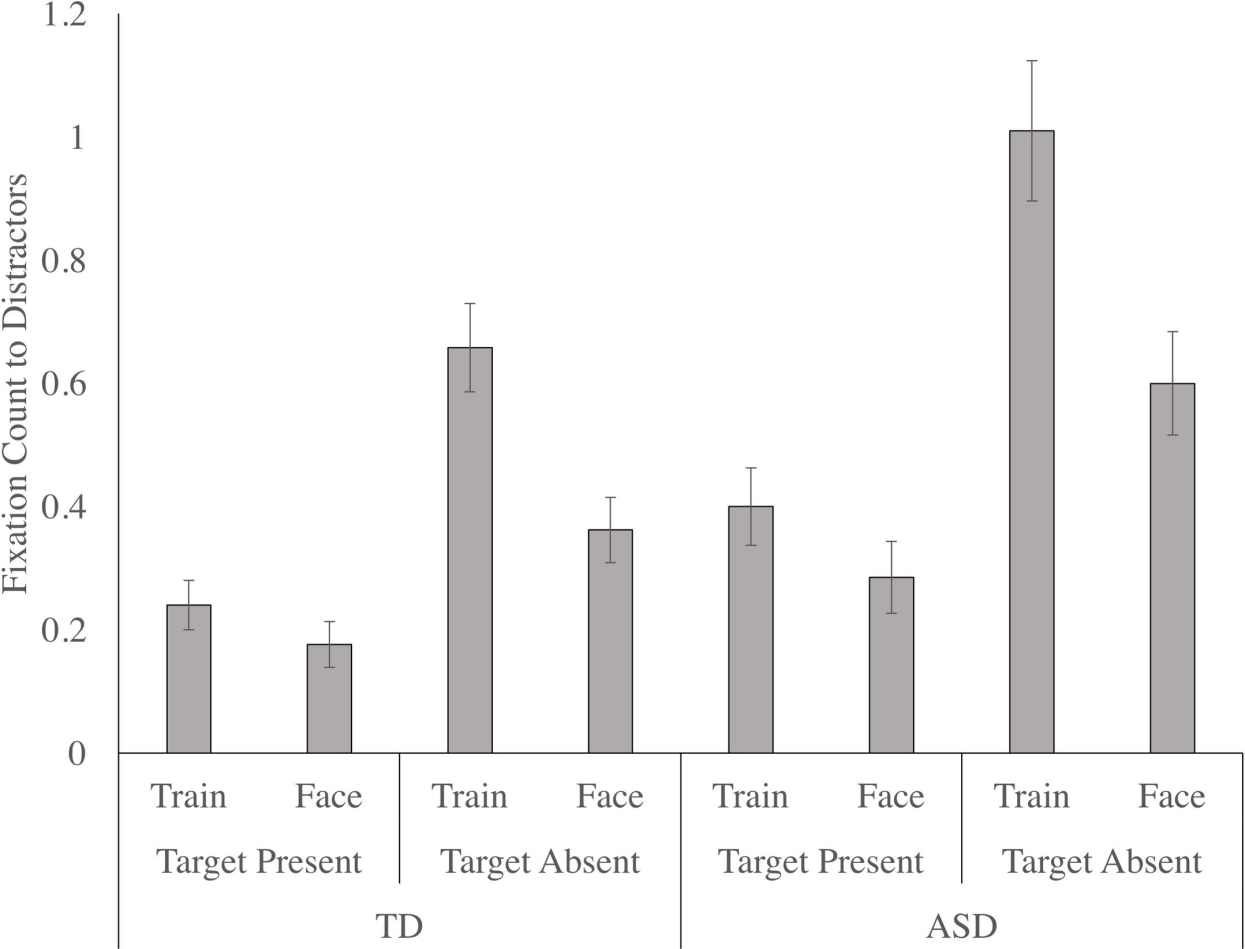
The number of fixations to the distractors (mean and standard error), for both the autistic and non-autistic children in the inverted condition as a function of target (present, absent) and distractor (train, face).

There was a significant main effect of target, F (1, 33) = 122.845, p < .001, n _p_^2^ = .788, as the critical distractor ROIs were fixated more when the target was absent, relative to when the target was present. The main effect of distractor condition was significant, F (1, 33) = 44.634, p < .001, n _p_^2^ = .575, as participants fixated the critical distractor more in the train condition, relative to the face condition. The target by distractor interaction was significant, F (1, 33) = 44.942, p < .001, n _p_^2^ = .577, with paired samples t-tests confirming that train distractors were fixated more than face distractors on target present, t (34) = 2.839, p = .008, d = 0.40, and target absent, t (34) = 8.002, p < .001, d = 0.97, trials; however the difference was more pronounced when the target was absent (see Fig 8). There was a main effect of group, F (1, 33) = 6.674, p = .014, n _p_^2^ = .168, as overall, autistic children fixated the critical distractor ROIs more than the non-autistic children. The target by group, F (1, 33) = 5.414, p = .026, n _p_^2^ = .141, interaction was also significant. Paired samples t-tests indicated that critical distractors were fixated more on target absent trials than target present trials for non-autistic, t (24) = 7.519, p < .001, d = 1.76, [1.17, 2.52], and autistic, t (7) = 6.561, p < .001, d = 2.39, [1.32, 4.26], groups; however, the difference was more pronounced in the autistic children relative to the non-autistic children (see Fig 8). The distractor by group and the target by distractor by group interactions were not significant.

## 7. Discussion eye-tracking results

A subset of participants’ eye-movements were examined in order to investigate whether faces and trains had differential effects on overt attention. That is, while the task was to manually detect the presence or absence of a target (a butterfly), we did not instruct participants to maintain central fixation, and so we expected that most participants would move their eyes around the display in search of the target. We quantified the number and timing of fixations to the butterfly target and the critical train and face distractors. The butterfly target was fixated on the majority of target present trials. In the upright condition, distractor condition (train, face, neutral), did not influence the number, or timing, of fixations to the target. However, in the inverted stimulus condition, the target was fixated less frequently, and later, in the train distractor condition relative to the neutral condition. Faces were found to only have a marginal effect on first fixation time to the target, relative to the neutral condition, and faces had no effect on the number of fixations to the target. However, this effect of distractor condition was driven primarily by the autistic children. When tested separately, only the autistic children, but not the non-autistic children, showed an effect of distractor class on first fixation time to the butterfly target.

In both the upright and inverted stimulus conditions, train and face distractors, respectively, were fixated at least once on fewer than half of the trials. This result was not unexpected, as when Devue and colleagues (2012) investigated oculomotor capture by task irrelevant distractors, they found that the distractors only captured eye movements on between 8.5 and 13.12% of trials. Despite the small number of trials the critical distractors were fixated, relative to the target butterfly, it is still interesting to note that train distractors were fixated on almost twice as many trials as face distractors. In addition, when the target was absent, the distractors were first fixated later, but fixated more often. Paralleling this result, when examining fixation counts (i.e. number of fixations averaged across trials) train distractors were fixated more overall than face distractors, and this effect was larger in the target absent conditions.

Across both upright and inverted conditions, autistic children fixated the distractors more frequently than non-autistic children. In the upright condition, although train distractors were first fixated earlier than face distractors, an interaction between distractor and group indicated that this effect was driven by the autistic children, as they first fixated the train distractors earlier than the face distractors, an effect that was not seen in the non-autistic children. In the inverted condition, autistic children fixated the train distractors earlier than the face distractors when the target was present, but showed no difference in first fixation latency when the target was absent. Whereas, non-autistic children showed the opposite pattern, first fixating on train distractors earlier than face distractors when the target was absent, but did not show a difference in first fixation latency when the target was present.

Similar to the manual response data, the eye-tracking data indicates that train distractors had a larger effect on overt attention to the target, and were first fixated earlier, and more often, than face distractors, particularly in the inverted condition. When considering group differences, we find that non-autistic children had faster manual target detection RTs, and were also faster to first fixate the target butterfly. However, the attention capture (manual RT) effect for trains that we observed in non-autistic children was not paralleled by fewer fixations to, or later first fixations to, the target in the presence of train distractors. Thus, it appears that the manual and eye-tracking data may be indexing different aspects of how the attention system behaves in the presence of critical stimuli (e.g., see [29]).

Previous research has suggested that faces have a special status in the attentional system of humans [5–8], yet we found that trains had a larger effect on responses (both manual and eye-tracking) than faces. We wondered whether one explanation might be that the train distractors had a higher affective value than faces for children. This idea was explored in Experiment 2.

## 8. Experiment 2

Autistic individuals have demonstrated greater exploration and perseveration on particular classes of objects, like trains, over faces [41,46]. This preference for circumscribed interest objects was suggested to involve an increased reward value relative to faces for autistic individuals [41]. Also, heightened affective responses to certain classes of circumscribed interest objects is suggested to result in an increase in the salience of these objects for autistic individuals [57]. In order to investigate whether the attention capture by trains in Experiment 1 might be related to the affective value of the stimuli, we had a separate group of autistic and non-autistic children rate the valence and arousal of the experimental stimuli.

## 9. Experiment 2 methods

### 9.1 Participants

Thirty-five autistic and non-autistic participants between the ages of 6 and 12 years participated in this experiment. Children were matched on age, but the non-autistic group had a higher average IQ (see Table 1). To ensure the difference in IQ did not influence the findings, we initially matched the samples on IQ (autistic n = 13, IQ: M = 101.62; SD = 14.66, non-autistic n = 13 IQ: M = 110.08, SD = 5.63, t(24) = 1.943, p = .071, d = 0.76). However, because the results remained the same as with the full sample, we opted to include all participants as it was clear that the difference in IQ was not influencing the observed results.

### 9.2 Apparatus and stimuli

Participants completed a picture rating task programmed using Qualtrics Software (Qualtrics, Provo, UT), while seated at a desktop computer with a 17” monitor. Experimental stimuli were the train, faces, and neutral objects used in Experiment 1. Two versions of the experiment were created. The first version had 4 stimuli from each of the stimulus categories (fruit, flower, chair, clock, fish, face, and train), whereas the second version had the remaining 4 stimuli from each category. Participants from each group, autistic and non-autistic, were randomly assigned to one of the two versions.

### 9.3 Procedure

Children participated in this experiment in small groups. The children were brought to a computer lab where each child was assigned a computer workstation. The workstations were organized in such a way that the children were not able to view another child’s computer screen while performing the task.

**Fig 9:**
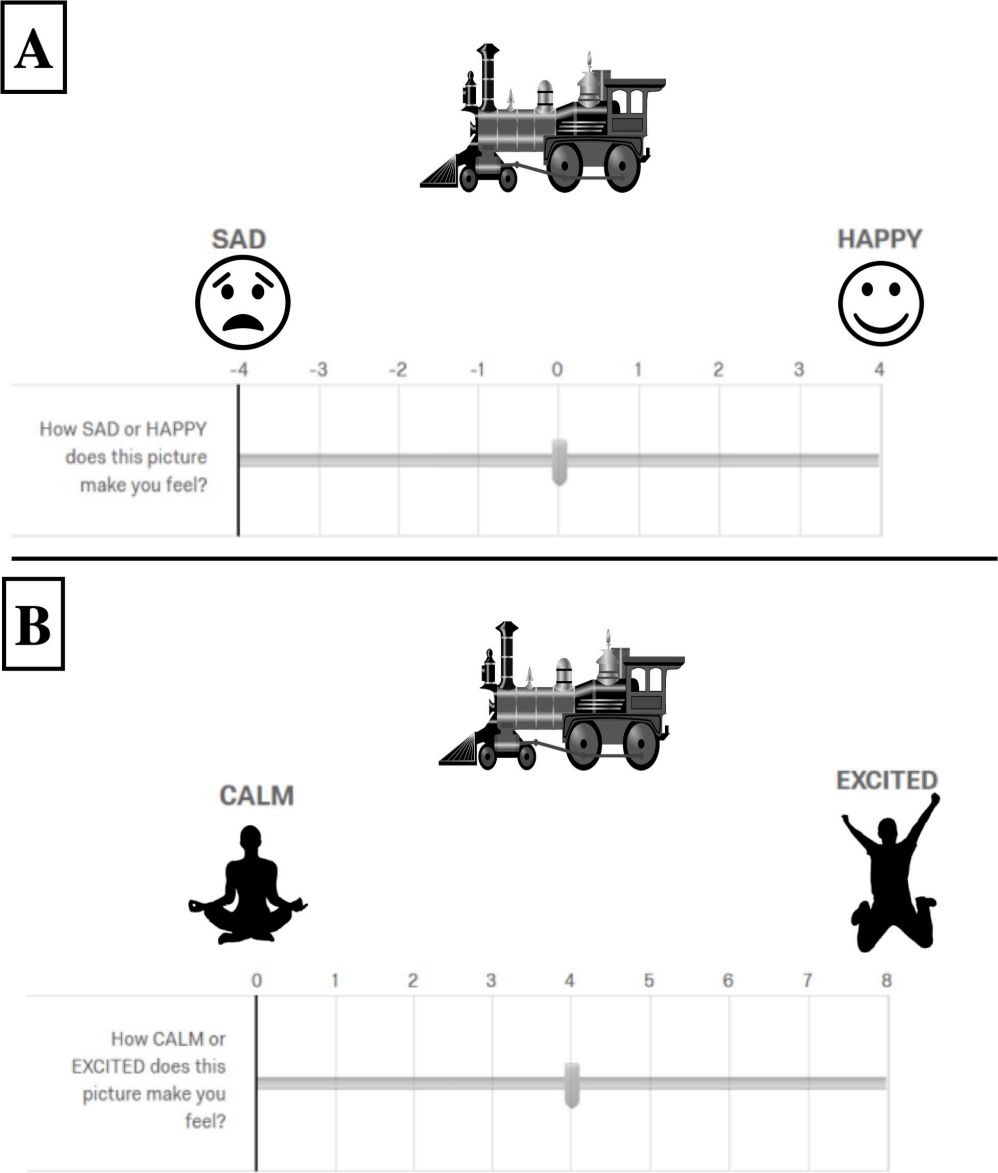
Experiment 2 procedure. A: Participants rated the valence of the stimuli by indicating how sad versus happy each stimulus made them. Valence ratings could vary from -4 (sad) to +4 (happy). B: Participants rated their level of arousal in response to the stimuli by indicating how calm versus excited each stimulus made them. Arousal ratings could vary from 0 (calm) to 8 (excited). The trials were self-paced. Note: Due to copyright restrictions, many of the images in this figure are representative. See S1 File for information on how to access the actual images used in the experiment.

On each trial the child was randomly presented with 1 of 32 experimental stimuli. Below each stimulus a slider appeared to rate either valence or arousal. For each stimulus the participant first rated the valence, then proceeded to the next trial where they would rate the arousal for the same image. The valence slider ranged from -4 to +4. Above -4, a frowning emoji and the word “SAD” appeared. Above the +4, a smiling emoji and the word “HAPPY” appeared. The arousal slider ranged from 0 to 8. Above the 0, a calm looking Snoopy (Copyright 2018 Peanuts Worldwide, LLC) cartoon and the word “CALM” appeared. Above the 8, a dancing Snoopy cartoon (Copyright 2018 Peanuts Worldwide, LLC) and the word “EXCITED” appeared (see Fig 9). While the stimuli were randomized across the task, the valence and arousal ratings were paired for each stimulus, so they always occurred one after another.

Once seated at a computer, the children were asked if they would like to play a picture rating game, and were instructed that if they no longer wanted to play, they could stop at any time. The experimenter then read a script to the children that stated: “We are now going to look at pictures and decide how happy verses sad they make us feel, and how calm verses excited they make us feel.” The children were then walked through a practice trial where a picture of a ladybug appeared on their screen and the experimenter said: “When I look at this ladybug, I get a little bit sad, because I don’t like bugs near me. So I am going to slide this bar closer to the sad face. BUT, since it only makes me a little bit sad, I am not going to slide it all of the way. If I was really sad, then I would slide it all the way. Just like if it made me feel a bit happy I would move the bar closer to the happy face–and if it made me REALLY REALLY happy, because I love ladybugs, I would move it all the way over to the happy face. If I am equally sad and happy I would leave it in the middle. Next I need to judge how calm verses excited this ladybug makes me feel. So if I feel very calm and relaxed while looking at the ladybug then I would drag the bar closer to calm Snoopy. BUT if I am feeling very energetic or excited seeing the ladybug, then I would move the bar closer to excited Snoopy. For me, the ladybug makes me a bit nervous, so I am going to leave the red button right in the middle–because I am not calm, but I am not super energetic either.” The trials were self-paced, and the children could take as long as they wanted to make their ratings. Most children took about 10 minutes to complete the task.

### 9.4 Statistical analysis

Mean valence and arousal ratings were calculated for the face, train, and neutral object stimuli for each child. A two-way RM-ANOVA was conducted on both valence and arousal ratings to determine whether they differed across stimulus category (train, face, neutral) and participant group (autistic, non-autistic). Prior to conducting this analysis, a preliminary analysis was conducted with version as a factor. Given no effects of version were found, data was collapsed across the two versions.

## 10. Experiment 2 results

### 10.1 Valence

A two-way RM-ANOVA was conducted to investigate the influence of stimulus category (train, face, neutral) and group (autistic, non-autistic) on valence judgements. There was a significant main effect of stimulus category, F (2, 66) = 25.630, p < .001, n _p_^2^ = .437, as face stimuli (M = -1.187, SE = .387) were rated significantly lower (less happy) than both train (M = 1.684, SE = .333; p < .001) and neutral (M = .945, SE = .228, p < .001) stimuli. Train stimuli were also rated higher on valence than the neutral stimuli (p = .045). The main effect of group, F (1, 33) = .338, p = .565, n _p_^2^ = .010, and the stimulus category by group interaction, F (2, 66) = .106, p = .899, n _p_^2^ = .003, were both nonsignificant (see Table 2).

**Table 2:** Mean (standard deviation) ratings of valence and arousal for Experiment 2.

	Valence			Arousal		
	Neutral	Face	Train	Neutral	Face	Train
Autistic	.86 (1.48)	-1.24 (2.52)	1.45 (2.24)	3.44 (1.99)	2.42 (2.41)	4.91 (2.44)
Non-Autistic	1.04 (1.19)	-1.14 (2.03)	1.92 (1.62)	4.12 (1.75)	2.42 (2.22)	5.39 (2.11)

### 10.2 Arousal

A two-way RM-ANOVA was conducted to investigate the influence of stimulus category (train, face, neutral), and group (autistic, non-autistic), on arousal judgements. There was a significant main effect of stimulus category, F (2, 66) = 18.696, p < .001, n _p_^2^ = .362, as face stimuli (M = 2.423, SE = .392), were rated significant as less arousing than both train (M = 5.150, SE = .386; p < .001), and neutral (M = 3.781, SE = .386, p = .018), stimuli. Train stimuli were rated higher on arousal than neutral stimuli (p = .001). The main effect of group, F (1, 33) = .560, p = .460, n _p_^2^ = .017, and the stimulus category by group interaction, F (2, 66) = .311, p = .734, n _p_^2^ = .009, were not significant (see Table 2).

## 11. Experiment 2 discussion

To investigate whether the attention capture by trains observed in the children in Experiment 1 was related to the affective value of the stimuli, we had a separate group of similarly aged children rate the valence and arousal of the stimuli. The results of Experiment 2 indicate that both autistic and non-autistic children reported that the train stimuli made them significantly happier and more excited than faces and other types of distractors.

## 12. General discussion

The goal of this research was to examine potential attentional biases to face and train objects in autistic and non-autistic children. In Experiment 1, both autistic and non-autistic children were more accurate, but slower, at determining the absence or presence of the butterfly target when the target was absent. This slower search time in the target absent condition was expected and likely reflects a serial-search strategy, whereby each item was inspected before a response was given, meaning more items were inspected in the target absent condition, relative to the target present condition where the search was terminated once the butterfly was observed.

Importantly, the different classes of distractor stimuli did not influence the accuracy of target identification. However, when comparing manual target detection times on trials where a face, train, or only neutral distractors were present, both trains and faces were found to slow the performance of both the autistic and non-autistic children, with trains having a marginally larger effect on attention capture than faces. Thus, in contrast to previous research [e.g., 25–27] suggesting that faces are a special class of stimuli, we found only weak evidence that faces captured attention involuntarily, with train stimuli capturing children’s attention just as much, if not more, than faces.

Past research that compared search performance across upright and inverted conditions, has found a face-inversion effect(e.g. [5,7]), whereby task-irrelevant faces slow RT when presented in an upright orientation, but not in an inverted orientation. Previous research has demonstrated that when processing faces, adults tend to be particularly sensitive to the spatial relations between the different parts of the face, or their configural relations [14,17,21]. Inverting the face is thought to prevent configural processing [48,49], which means the individual must rely on low-level features that remain unchanged when the face is inverted (e.g. contrast, edge density, etc.; [7]). We did not find an interaction between distractor type and orientation, and thus, we did not replicate this face-inversion effect within the present attention capture task. One explanation for a lack of inversion effect in the current study is the young age of the children. It has previously been demonstrated that for perceptual tasks, the face inversion effect becomes stronger with age, with 7 year olds failing to demonstrate an inversion effect, 10 year olds showing a small inversion effect, and adults showing a strong inversion effect [58]. Given many of the children in this study were under the age of 10 it is possible that these children have yet to adopt configural processing strategies, thus the inversion of the stimuli did not influence their attentional capture. This would also explain the difference between our findings and that of Langton and colleagues [7]. While Langton and colleagues [7] used the same stimulus set as the present study, they studied adult participants, who are more likely to use configural processing strategies, and thus show an inversion effect. With that in mind, we did find that both faces and trains captured attention relative to the neutral stimuli, suggesting that both face and train stimuli were prioritized by the attentional systems of both the autistic and non-autistic children. This attentional bias, without an inversion effect, may suggest that while attentional biases develop early on, neural specialization for stimuli (evidenced by an inversion effect), develops later on. It may be that the later-developing neural specialization for faces is required for attention to be *disproportionately* allocated to (particularly upright) faces relative to other non-social stimuli like trains. Our findings, and those of others, underscore the importance of studying the typical and atypical development of attention biases [40,41,50] and neural specialization [38,39] for *both* social and non-social stimuli.

Although the manual RT data revealed few attention capture effects, the eye tracking data revealed slightly different patterns worthy of discussion. For these analyses, because of unbalanced completion of upright and inverted blocks across participants, we analyzed the conditions separately. For the upright condition, fixation count and first fixation time to the target butterfly ROI were not influenced by the different classes of distractor stimuli, suggesting that task-irrelevant trains and faces did not differentially capture overt attention away from the target. However, children first fixated the train earlier, and committed more fixations to the train, relative to the face, particularly when the target was absent. When broken down by group, autistic participants fixated the train earlier than the face ROI, while train and face ROIs were first fixated at similar latencies for the non-autistic children. Thus, the eye-tracking data for the upright condition indicated a stronger interest in trains relative to faces in autistic children.

For the inverted condition, the target butterfly was fixated less frequently in the presence of train distractors, relative to face distractors. The train distractor also slowed the first fixation to the butterfly, relative to the neutral condition, particularly for autistic children. These results suggest that the task-irrelevant train stimuli more strongly influenced overt attention than the task-irrelevant face stimuli. With regard to fixations occurring on the (inverted) critical distractors themselves (face, train), the distractors were fixated slower, but more often, when the target was absent. Children fixated the inverted train distractors more frequently than the inverted face distractors, with the autistic children also fixating the distractors more overall. The non-autistic children had similar first fixations latencies across distractor stimuli when the target was present, but first fixated the train faster than the face when the target was absent. Autistic children first fixated the train sooner than the face when the target was present, but had similar first fixation times across distractor stimuli when the target was absent. Thus, similar to the upright condition, we see that task-irrelevant train distractors appeared to more strongly drive oculomotor behavior than task-irrelevant face distractors, for both autistic and non-autistic children, although this varied depending on the presence of absence of the target.

To examine possible mechanisms driving stronger attention capture for trains than for faces, Experiment 2 had a separate group of similarly aged children rate the valence and arousal of all stimuli used in Experiment 1. The results indicated that autistic and non-autistic children reported that the train stimuli made them significantly happier and more excited than the face stimuli. There was no interaction with group, and therefore no evidence that autistic children found faces to be less interesting, relative to non-autistic children. Together with the results of Experiment 1, these findings suggest that attention capture may have been driven, in part, by voluntary, or top-down, attentional processes related to the children’s personal interest or affective response to the stimuli. However, we cannot rule out the possibility that low-level properties of the stimuli may have driven attentional capture to some extent, despite the trains and faces being matched on mean luminance contrast. Indeed, the role of low-level visual information is important to consider in light of our findings that the observed effects for trains occurred as, if not more, robustly in the inverted condition. Future research will directly manipulate the saliency of face and train distractors to tease out this possibility.

The results of this study suggest that the notion that faces have a special status in the attentional system of humans [5,10,19,59] would need to be qualified considering factors such as the age of the individual, current task priorities, previous experience, or an individual’s personal interest in a particular aspect of the object or environment [2,60,61]. Under certain conditions the attentional system may prioritize faces over nonsocial stimuli, however, the capture of attention by faces may not be as automatic and robust as previously suggested. For example, Pereira and colleagues (2019a) investigated attentional bias to faces vs. houses in a series of 4 experiments wherein stimulus factors, task properties, response conditions, and eye-movements were controlled. The results of these experiments provided no evidence for spontaneous attentional biasing to social stimuli over nonsocial stimuli.

Another consideration, is that overall accuracy was quite high, between 92 and 97%, and median target detection times were short (<2s) suggesting the task was quite easy. As a result, it is possible that the short amount of time spent viewing the stimuli on each trial made it difficult to determine how the different classes of distractors might have been prioritized by the attentional system, had they been presented for longer. The eye-tracking data demonstrates that the distractors were viewed on fewer than half of the trials, thus, this may have created inadequate power to detect differences in oculomotor biases. In future studies, increasing the difficulty of the task (e.g., making the distractors more visually similar to the target) could lead to more variable performance and more fixations, thereby making it easier to dissociate the relative influence of the different distractor classes on attention capture.

One possible explanation for the lack of disproportionate attention capture by faces reported in this study, relative to other studies (although [7] used the same faces), is the face stimuli utilized. The face stimuli had neutral expressions and were cropped to remove the external features (hair and ears). This choice of stimuli may have reduced their affective value, as demonstrated by the low valence and arousal ratings provided by the children in Experiment 2. While the low affective value of these stimuli certainly may have contributed to their lack of disproportionate attention capture, this would support the idea that the attentional prioritization of faces is sensitive to stimulus and task parameters, and is not a general phenomenon.

Another limitation that must be acknowledged is the relatively small sample of children that completed the eye-tracking component of the experiment, particularly in the autistic group. For this reason, the eye tracking results should be interpreted with caution. In addition, due to imbalanced completion of upright and inverted blocks, we were not able to perform the omnibus ANOVA with orientation as a factor, needed to probe for inversion effects on oculomotor behavior. However, given that the general patterns found in the oculomotor capture data closely mirrored the manual RT findings, which were based on a much larger sample, we believe that the reported findings are still a valid index of attentional prioritization in this population.

Lastly, response keys were not counterbalanced across subjects, thus it is possible that the faster manual RTs to target present trials in Experiment 1 may have been influenced by the hand used to indicate these responses.

## 13. Conclusion

In sum, our findings and that of others [29,30] suggest that factors such as stimulus, task parameters, and valence might play a stronger role in biasing attention to social stimuli than previously thought, particularly in children. Controversially, our findings suggest that it is not accurate to say that faces *generally/automatically* capture the attentional system of humans disproportionately more so than other nonsocial stimuli. Further research considering factors such as the age of the individual, current task priorities, previous experience, or an individual’s personal interest in a particular aspect of the object or environment is needed to more precisely pinpoint the development of attentional biases to faces and other objects [62].

## Supporting information

S1 File (DOCX)

S2 File (DOCX)
