# Supplementary material for: Attention capture by trains and faces in children with and without autism spectrum disorder
Source: PLoS One. 2021 Jun 18;16(6):e0250763. doi: 10.1371/journal.pone.0250763 (PMC8213190; doi:10.1371/journal.pone.0250763)
Supplement: S1 File — (DOCX) [file pone.0250763.s001.docx]

| Stimulus Name | Image | Description and Source |
| --- | --- | --- |
| bwface1 | 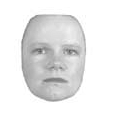 | Borrowed (with permission) from Langton, S. R. H., Law, A. S., Burton, A. M., & Schweinberger, S. R. (2008). Attention capture by faces. *Cognition*, *107*(1), 330–342. <http://doi.org/10.1016/j.cognition.2007.07.012>  Note: this image does not appear in the Langton publication, but was rather part of the stimulus set used in the experiment. |
| bwTarget1 | 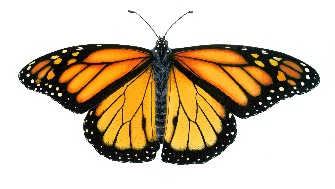 | Monarch (free)  https://pnghut.com/png/mgGECamTd1/monarch-butterfly-biosphere-reserve-sanctuary-pacific-grove-milkweed-transparent-png |
| bwTarget2 | 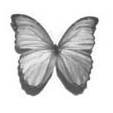 | Blue Morpho Butterfly  <https://commons.wikimedia.org/wiki/File:Blue_morpho_butterfly.jpg> |
| bwTarget5 | 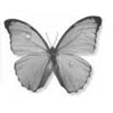 | Blue Butterfly (free)  http://clipart-library.com/clipart/6cpozqy9i.htm |
| bwTrain2 | 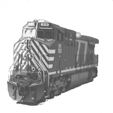 | 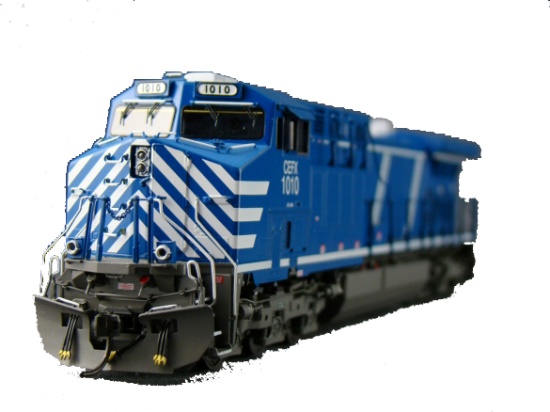https://www.brasstrains.com/BrassGuide/PDG/Detail/11437/HO-Diesel-CIT-GE-AC4400CW |
| bwTrain12 | 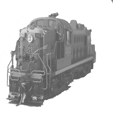 | 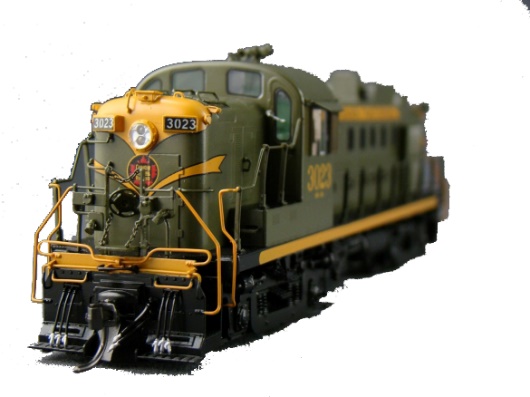  <https://www.brasstrains.com/BrassGuide/PDG/Detail/31879/HO-Diesel-Central-New-Jersey-ALCO-RS-3> |
| bwTrain14 | 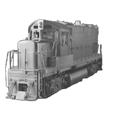 | 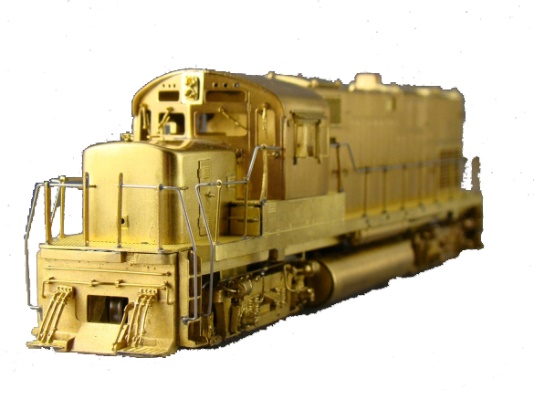<http://www.brasstrains.com/BrassGuide/PDG/Detail/14516/HO-Diesel-MISC-ALCO-C420> |
| chair1 | 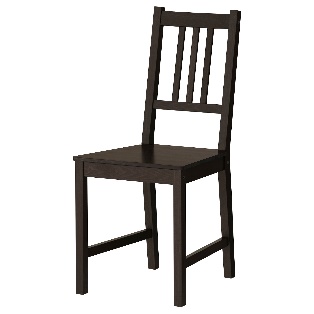 | Ikea STEFAN chair  <https://www.ikea.com/ca/en/p/stefan-chair-brown-black-00211088/> |
| chair 4 | 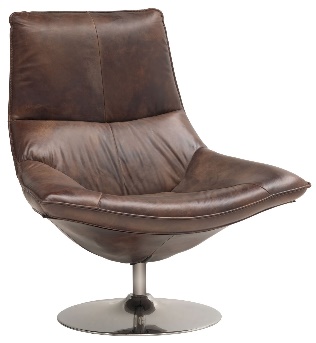 | Andrew Martin Carlotta Chair  <https://olivias.com/collections/armchairs/products/carlotta-chair> |
| chair 5 | 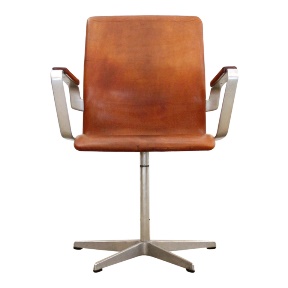 | Arne Jacobson Oxford Classic Low– can’t find exact source of image, but two very similar pictures:  <https://www.bukowskis.com/en/auctions/E250/lots/1027547-arne-jacobsen-an-office-chair-by-arne-jacobsen-oxford-low-fritz-hansen-21st-century>  <https://fritzhansen.com/en/products/chairs/3271c_oxfordclassic_leather> |
| chair 6 | 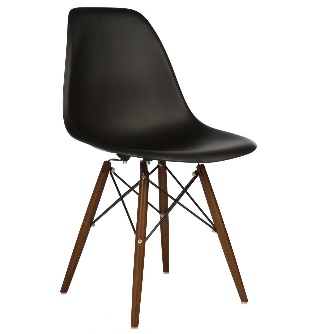 | Eames Chair (replica)  <https://www.mattblatt.com.au/mb/buy/matt-blatt-set-of-2-eames-premium-dsw-chair-replica-black-seat-walnut-legs-matt-blatt/> |
| chair 7 | 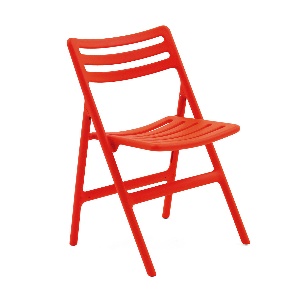 | MAGIS Folding Air-Chair  <https://www.masonionline.com/foldingairchair-sd75> |
| chair 8 | 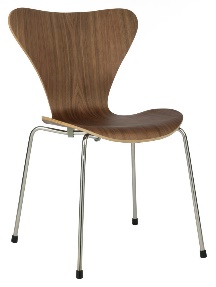 | “Seven” by Arne Jacobson and Fritz Hansen  <https://artorigo.com/furniture/aj-3107-seven-chair-in-rosewood/id-20672>  <https://godrie.eu/product/1955-arne-jacobsen-fritz-hansen-butterfly-chair-in-natural-cherry/> |
| clock 1 | 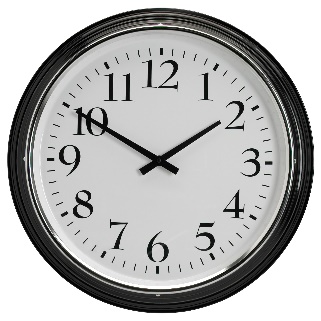 | IKEA Bravur  <https://www.ikea.com/ca/en/p/bravur-wall-clock-black-40391902/> |
| clock 5 | 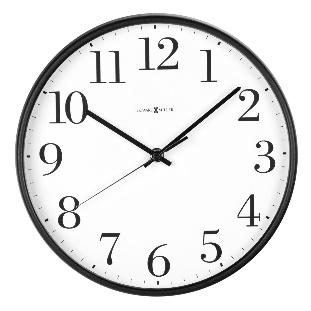 | Clock (stock photo is Free)  <http://clipart-library.com/clipart/riLo5kpoT.htm> |
| face3 | 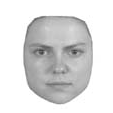 | Borrowed (with permission) from Langton, S. R. H., Law, A. S., Burton, A. M., & Schweinberger, S. R. (2008). Attention capture by faces. *Cognition*, *107*(1), 330–342. http://doi.org/10.1016/j.cognition.2007.07.012  Note: this image does not appear in the Langton publication, but was rather part of the stimulus set used in the experiment. |
| face5 | 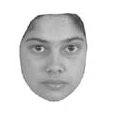 | Borrowed (with permission) from Langton, S. R. H., Law, A. S., Burton, A. M., & Schweinberger, S. R. (2008). Attention capture by faces. *Cognition*, *107*(1), 330–342. http://doi.org/10.1016/j.cognition.2007.07.012  Note: this image does not appear in the Langton publication, but was rather part of the stimulus set used in the experiment. |
| flower 3 | 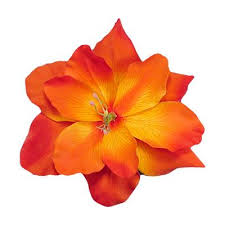 | Daisy (stock photo is free)  <https://pnghut.com/png/Bm4WNVdBTH/orange-cut-flowers-petal-common-daisy-flower-transparent-png> |
| flower 5 | 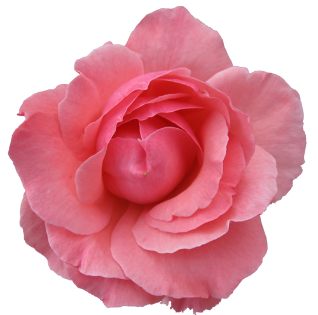 | Rose (stock photo is free)  <http://clipart-library.com/clipart/AibrneEeT.htm> |
| flower 7 | 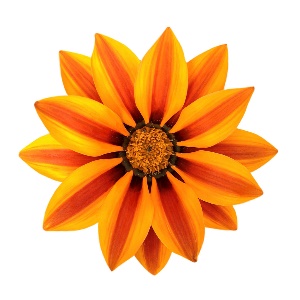 | Gazania (stock photo is free)  <http://www.pngmart.com/image/56490> |
| flower 8 | 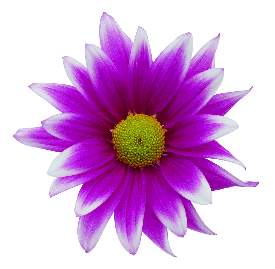 | Dahlia? (stock photo is free)  <http://clipart-library.com/clipart/kT85oeyxc.htm> |
| fruit 5 | 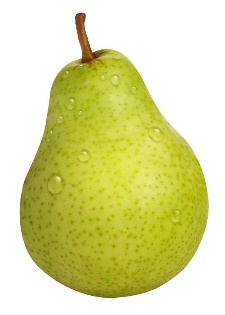 | Green Pear (stock photo is free)  <https://www.shutterstock.com/image-vector/realistic-green-pear-isolated-on-white-646906984> |
| fruit 7 | 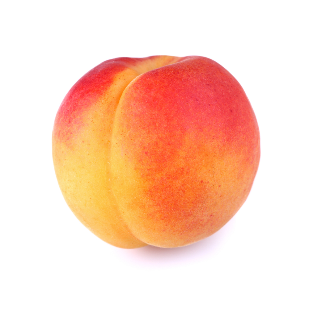 | Peach (stock photo is free)  <https://depositphotos.com/stock-photos/peach-fruit.html?qview=15258551> |
| fruit 8 | 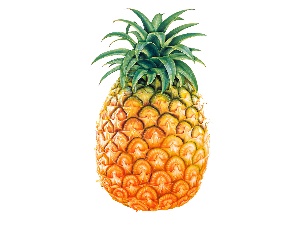 | Pineapple (stock photo is free)  <http://clipart-library.com/clipart/pineapple-clipart-9.htm> |
| target 8 | 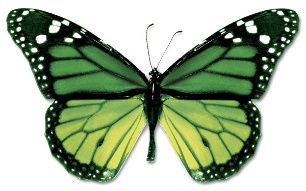 | Butterfly (stock photo is free)  <http://clipart-library.com/clipart/6ir5a6p4T.htm> |
| Happy Face | 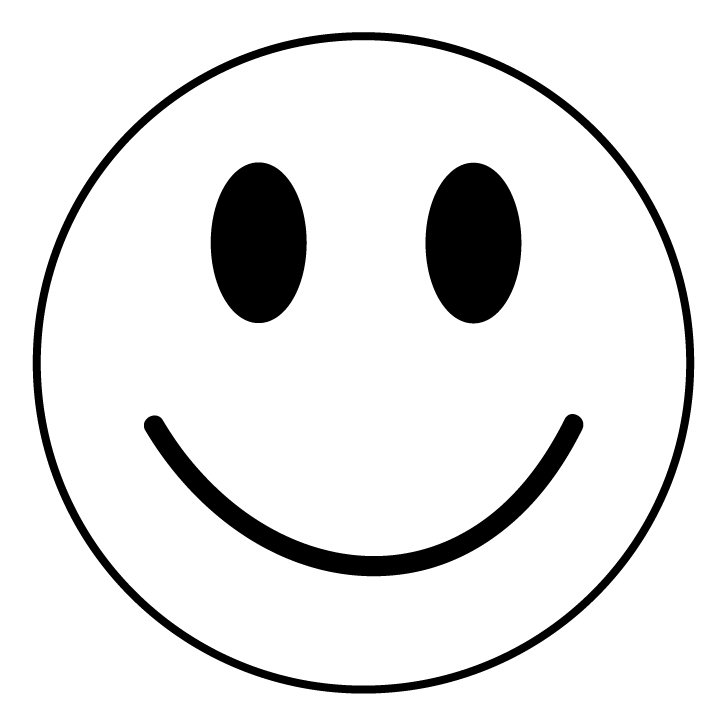 | Happy Face (free stock photo)  http://clipart-library.com/clipart/8gTeELpid.htm |
| Sad Face | 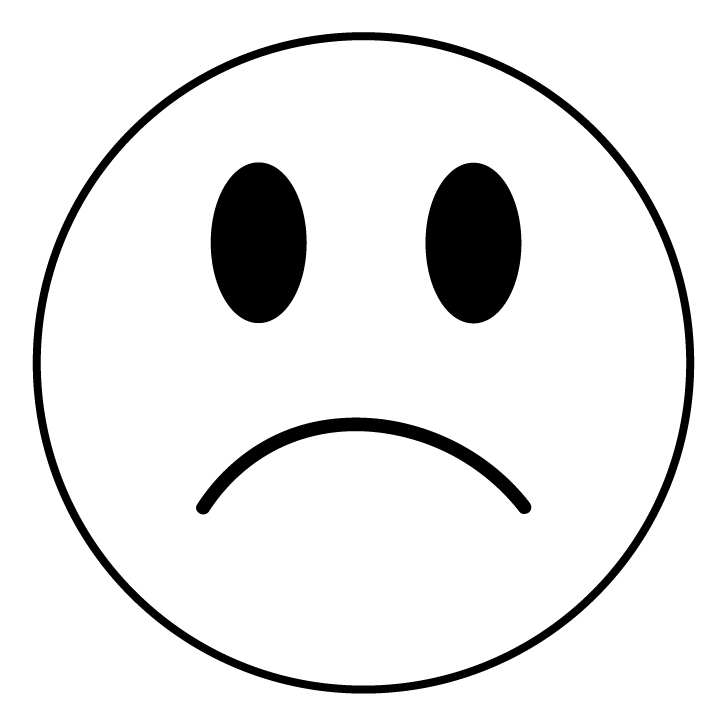 | Sad Face (free stock photo)  http://clipart-library.com/clipart/Xpio9LEcE.htm |
| Calm Person | 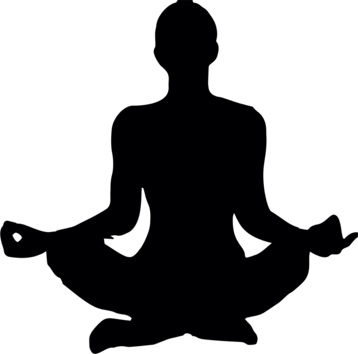 | https://www.iconspng.com/image/86361/female-yoga-pose-silhouette-13 |
| Excited Person | 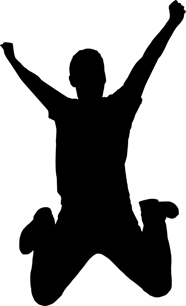 | https://pixabay.com/vectors/silhouette-expressions-happy-3127948/ |
