# Supplementary material for: Attention capture by trains and faces in children with and without autism spectrum disorder
Source: PLoS One. 2021 Jun 18;16(6):e0250763. doi: 10.1371/journal.pone.0250763 (PMC8213190; doi:10.1371/journal.pone.0250763)
Supplement: S2 File — (DOCX) [file pone.0250763.s002.docx]

**Supplementary Materials**

To take a closer look at the effect of the distractor conditions on accuracy and reaction time, we conducted the analyses separately for the upright and inverted conditions.

**Accuracy**

**Upright.** There was a significant main effect of target, F (1, 74) = 37.765, p < .001, n^2^ = .338, as accuracy was higher when the target was absent, relative to when the target was present. The main effect of distractor, F (2, 148) = 2.448, p = .090, n2 = .032, and group, F (1, 74) = .512, p = .476, n2 = .007, as well as the target by group, F (1, 74) = .263, p = .609, n2 = .004, distractor by group, F (2, 148) = .299, p = .742, n2 = .004, target by distractor, F (2, 148) = .998, p = .371, n2 = .013, and target by distractor by group, F (2, 148) = 1.460, p = .236, n2 = .019, interactions were not significant.

**Inverted.** There was a significant main effect of target, F (1, 73) = 59.444, p < .001, n2 = .449, as accuracy was higher when the target was absent, relative to when the target was present. The main effect of distractor, F (2, 146) = .040, p = .961, n2 = .001, and group, F (1, 73) = .947, p = .334, n2 = .013, as well as the target by group, F (1, 73) = .529, p = .469, n2 = .007, distractor by group, F (2, 146) = 1.191, p = .307, n2 = .016, target by distractor, F (2, 146) = 3.021, p = .052, n2 = .040, and target by distractor by group, F (2, 146) = .339, p = .713, n2 = .005, interactions were not significant.

**Median RT**

**Upright.** There was a significant main effect of target, F (1, 74) = 226.197, p < .001, n2 = .753, as RT was faster when the target was present, relative to when the target was absent. The main effect of group, F (1, 74) = 10.361, p = .002, n2 = .123, was also significant, as TD participants responded faster than ASD participants. The main effect of distractor, F (2, 148) = 2.916, p = .057, n2 = .038, was marginally significant, and the distractor by group, F (2, 148) = 1.083, p = .341, n2 = .014, and target by distractor, F (2, 148) = 1.373, p = .257, n2 = .018, interactions were not significant. However, the target by group interaction was significant, F (1, 74) = 7.084, p = .010, n2 = .087, reflecting that on average TD participants were 192.18 ms slower when the target was absent relative to when the target was present, while on average the ASD participants were 274.82 ms slower when the target was absent relative to when the target was present. The target by distractor by group, F (2, 148) = 3.575, p = .030, n2 = .046, interaction was also significant. To further explore this interaction, two-way RM-ANOVAs were conducted to investigate the influence of the target and distractor conditions on RT for each of the two groups. For the ASD group, the main effect of target was significant, F (1, 27) = 85.184, p < .001, n2 = .759, as RT was faster when the target was present, relative to when the target was absent. However, the main effect of distractor, F(2, 54) = 1.286, p = .285, n2 = .045, and the target by distractor interaction, F (2, 54) = 2.005, p = .145, n2 = .069, were not significant. For the TD group, the main effect of target was significant, F (1, 47) = 140.878, p < .001, n2 = .750, as RT was faster when the target was present, relative to when the target was absent. The main effect of distractor was also significant, F (2, 94) = 3.164, p = .047, n2 = .063. Pairwise comparisons indicated that RT was slower in the presence of trains, relative to neutral (p = .026), but not face (p = .335) distractors. Face distractors did not have a significant effect on RTs relative to neutral distractors (p = .116). The target by distractor interaction was also marginally significant, F(2, 94) = 2.893, p = .074, n2 = .058, as the effect of the distractors was larger in the target absent condition. Thus, for the TD group, trains, but not faces, captured attention; and for the ASD group, none of the critical distractors led to an attention capture effect.

**Inverted.** There was a significant main effect of target, F (1, 73) = 207.520, p < .001, n2 = .740, as RT was faster when the target was present, relative to when the target was absent. The main effect of group, F (1, 73) = 8.839, p = .004, n2 = .108, was also significant, as TD participants responded faster than ASD participants. The target by group interaction was significant, F (1, 74) = 8.191, p = .005, n2 = .101, as on average TD participants were 215.21 ms slower when the target was absent relative to when the target was present, while on average the ASD participants were 321.93 ms slower when the target was absent relative to when the target was present. The main effect of distractor, F (2, 146) = 6.950, p = .001, n^2^ = .087 was significant, as across groups RT was slower in the presence of train distractors, relative to neutral (p < .001) and face (p = .029) distractors. RTs were similar in the presence of the face and neutral distractors (p = .228). The distractor by group, F (2, 146) =.401, p = .671, n2 = .005, and target by distractor by group, F (2, 146) = .095, p = .909, n2 = .001, interactions were not significant. However, the target by distractor, F (2, 146) = 6.578, p = .003, n^2^ = .083, interaction was significant. To explore this interaction, one-way RM-ANOVAs were conducted to investigate the influence of the distractors on RTs in both the target present and target absent conditions. For the target absent condition, there was a main effect of distractor, F (2, 148) = 9.197, p = .001, n2 = .111, as both train (p < .001) and face (p = .051; marginal) distractors slowed RTs, relative to neutral distractors. Train distractors also had a larger effect on RT relative to face distractors (p = .038). For the target present condition, the main effect of distractor, F (2, 148) = .327, p = .722, n2 = .007, was not significant.
